# KCNE1 induces fenestration in the Kv7.1/KCNE1 channel complex that allows for highly specific pharmacological targeting

**DOI:** 10.1038/ncomms12795

**Published:** 2016-10-12

**Authors:** Eva Wrobel, Ina Rothenberg, Christoph Krisp, Franziska Hundt, Benjamin Fraenzel, Karina Eckey, Joannes T. M. Linders, David J. Gallacher, Rob Towart, Lutz Pott, Michael Pusch, Tao Yang, Dan M. Roden, Harley T. Kurata, Eric Schulze-Bahr, Nathalie Strutz-Seebohm, Dirk Wolters, Guiscard Seebohm

**Affiliations:** 1 Department of Cardiovascular Medicine, Institute for Genetics of Heart Diseases (IfGH), University Hospital Muenster, Muenster D-48149, Germany; 2 Department of Analytical Chemistry, Ruhr University of Bochum, Universitaetsstrasse 150, Bochum 44801, Germany; 3 Project Management Office, Janssen Research & Development, a Division of Janssen Pharmaceutica N.V., 2340 Beerse, Belgium; 4 Safety Pharmacology Research, Translational Sciences, Janssen Research & Development, a Division of Janssen Pharmaceutica N.V., 2340 Beerse, Belgium; 5 Institute of Physiology, Ruhr University of Bochum, Universitaetsstrasse 150, Bochum 44801, Germany; 6 Istituto di Biofisica CNR, Via De Marini 6, Genova I-16149, Italy; 7 Departments of Medicine, Vanderbilt University School of Medicine, Nashville, Tennessee, USA; 8 Departments of Medicine, Pharmacology and Biomedical Informatics, Vanderbilt University School of Medicine, Nashville, Tennessee, USA; 9 Department of Pharmacology, University of Alberta, 9-70 Medical Sciences Building, Edmonton, Alberta, Canada T6G 2R3; 10 Interdisciplinary Centre for Clinical Research (IZKF), Faculty of Medicine, University of Münster, Münster D-48149, Germany

## Abstract

Most small-molecule inhibitors of voltage-gated ion channels display poor subtype specificity because they bind to highly conserved residues located in the channel's central cavity. Using a combined approach of scanning mutagenesis, electrophysiology, chemical ligand modification, chemical cross-linking, MS/MS-analyses and molecular modelling, we provide evidence for the binding site for adamantane derivatives and their putative access pathway in Kv7.1/KCNE1 channels. The adamantane compounds, exemplified by JNJ303, are highly potent gating modifiers that bind to fenestrations that become available when KCNE1 accessory subunits are bound to Kv7.1 channels. This mode of regulation by auxiliary subunits may facilitate the future development of potent and highly subtype-specific Kv channel inhibitors.

## Abstract (web summary)

Specificity of inhibitors of voltage-gated ion channels is crucial for their use as therapeutics. Here, the authors show that adamantane derivatives interact with a specific binding site on fenestrations that only become available when accessory subunits are bound to the channel.

## Graphical abstract

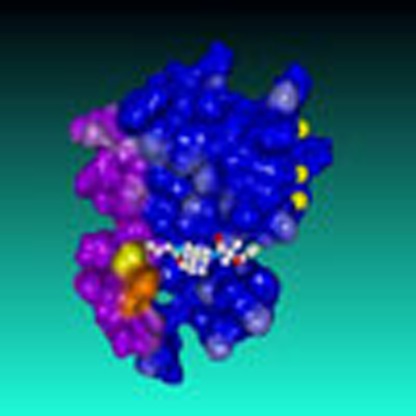

Voltage-gated potassium (Kv) channels enable the rapid, selective and passive transport of potassium ions through cellular membranes that regulate physiological processes such as ion-coupled transport, hormone secretion, vesicle cycling and cell excitability. Dysfunction of Kv channels causes numerous inherited or acquired channelopathies, and these channels are under investigation as potential therapeutic targets for acquired disease such as cardiac arrhythmia, neurodegenerative diseases and diabetes^1^,^2^,^3^,^4^,^5^,^6^,^7^,^8^. Kv channel diversity is impressive and is enhanced by the large number of different α-subunits, alternative splicing, post-transcriptional modifications and coassembly of similar but not identical pore forming α-subunits and/or accessory β-subunits to form heteromeric channels^9^,^10^,^11^. β-subunits modify the pharmacology, subcellular localization, gating and ion selectivity of Kv channels^12^,^13^,^14^,^15^,^16^. For example, KCNE1 β-subunits coassemble with Kv7.1 α-subunits to increase current magnitude, slow the rate of activation and remove apparent inactivation gating^17^,^18^,^19^.

The design of small compound inhibitors of voltage-gated channels with high affinity and subtype specificity has been particularly challenging. Most known small-molecule pore blockers of Kv channels bind to specific residues that line the wall of the central cavity^20^,^21^,^22^,^23^,^24^. With few exceptions^25^,^26^, these crucial residues are conserved in most K^+^ channels, complicating the discovery and development of subtype-specific channel inhibitors. Highly potent and selective peptide inhibitors (for example, natural toxins) that bind to a site outside the central cavity (for example, to the outer vestibule) are of limited practical use as therapeutic agents because they require parenteral administration and often have serious undesirable side effects^8^,^25^,^27^. Investigating the molecular basis of drug binding is also hampered by complicating issues of allosteric effects and studies are often limited to investigating the effects of point mutations on functional measures of drug effects, without directly assessing the site of drug binding.

Here we use multiple complementary approaches to characterize the binding mode of adamantane derivatives that can explain why these compounds are potent inhibitors of Kv7.1/KCNE1 channels. In addition to a conventional mutagenesis-based investigation of drug effects, we have generated an adamantane analog with a cross-linking moiety that allows direct mapping of its binding to specific channel peptide segments. Our findings suggest that these adamantanes bind with nanomolar affinity to fenestrations in the Kv7.1 channel that only form when the channel is in a complex with KCNE1 β-subunits. The mechanism of allosteric inhibition described here provides new opportunities for developing small-molecule inhibitors of heteromeric channels with the desired properties of very-high affinity and specificity.

## Results

### KCNE1 induces sensitivity of Kv7.1 to inhibition by AC-1

**Figure 1:**
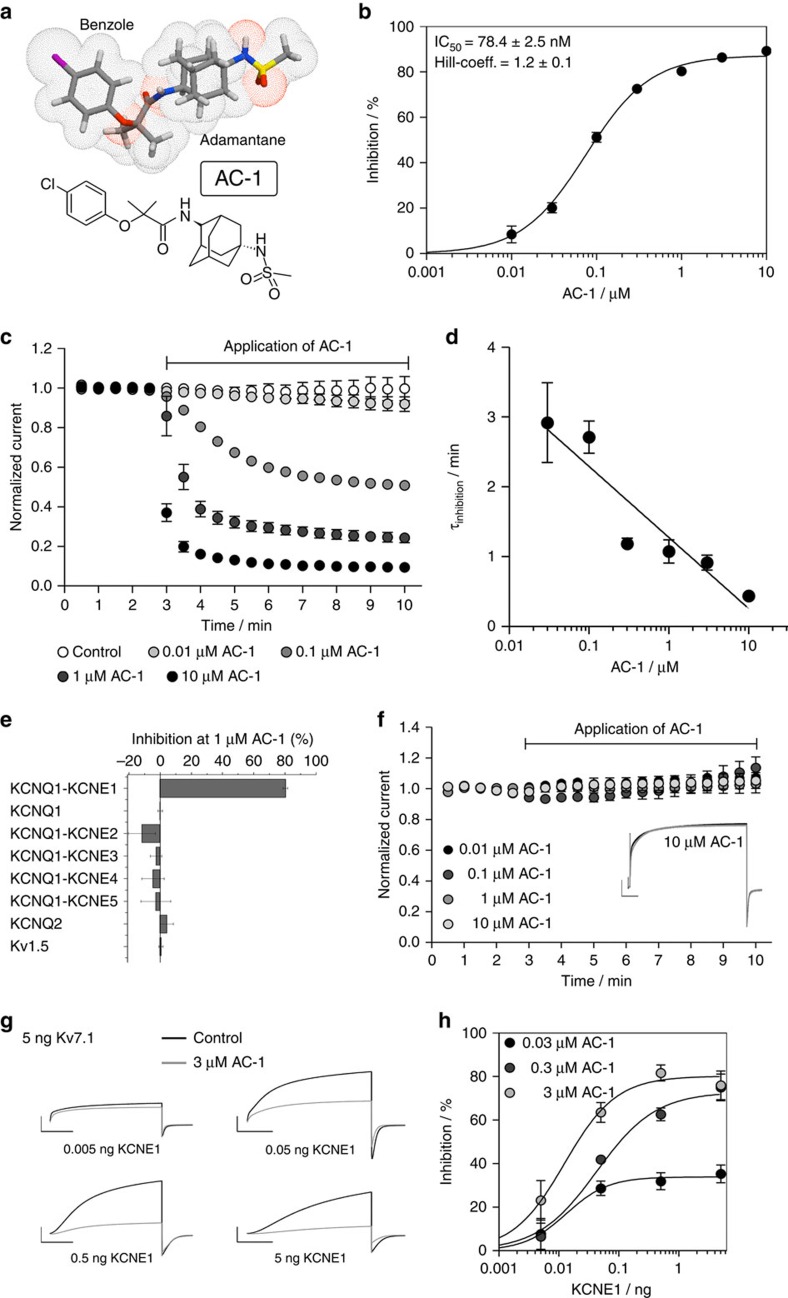
AC-1 inhibits Kv7.1 channels only in the presence of KCNE1. (**a**) Structure of adamantane compound AC-1. (**b**) Concentration-response relationship for inhibition of Kv7.1/KCNE channel currents by AC-1. Kv7.1/KCNE1 channel currents were activated by repetitive 7 s pulses to +40 mV applied from a holding potential of −80 mV. Inhibition was determined as percent change in current amplitude at the end of the depolarizing test pulse to +40 mV. Data were fitted (smooth curve) to the Hill equation (*n*=5–70,±s.e.m.). (**c**) The inhibitory effect of different AC-1 concentrations on Kv7.1/KCNE1 channels was determined by repetitive 7 s pulses to +40 mV applied from a holding potential of −80 mV. Current amplitudes at the end of the depolarizing test pulse were normalized to initial values and plotted against time (*n*=3–5, ±s.e.m.). (**d**) The onset of inhibition was described using a single exponential function and the time constant *τ*_inhibition_ was plotted against the respective AC-1 concentration. This relationship could be described using a linear function with *m*=−1.02±0.19 (*n*=3–5, ±s.e.m.). (**e**) Lack of 1 μM AC-1 inhibition of Kv7.1, Kv7.1/KCNE2–5, Kv7.2 and Kv1.5. (**f**) Lack of effect of different AC-1 concentrations on Kv7.1 homotetramers (*n*=3–5, ±s.e.m.). Example of homomeric Kv7.1 channel currents before (black) and after application (grey) of 10 μM AC-1 shown in the inlay. Currents were elicited by 7 s test pulses to +40 mV (scale bars indicate 1 μA, 1 s). (**g**) Inhibitory effects of 3 μM AC-1 on Kv7.1/KCNE1 channels is dependent on the level of KCNE1 expression. Currents were elicited as described in panel c (scale bars indicate 2 μA, 2 s). (**h**) The effect of variable KCNE1 expression on the sensitivity of Kv7.1 channels to three concentrations of AC-1. Inhibition of current was determined as described above and plotted against the amount (in ng) of cRNA injected into individual oocytes. Relationships were fitted using the Hill equation (smooth curves, *n*=3, ±s.e.m.).

Compounds binding to the central cavity of Kv7.1 have been reported to act on both homomeric Kv7.1 and heteromeric Kv7.1/KCNE1 channels, albeit with varying potency^20^,^21^,^28^,^29^. The adamantane compound AC-1 (2-(4-chlorophenoxy)-2-methyl-*N*-[5-[(methylsulfonyl)amino]tricyclo[3.3.1.13,7]dec-2-yl]-propanamide^30^, also known as JNJ303 (ref. 31) (Fig. 1a), inhibits heteromeric Kv7.1/KCNE1 channel complexes in a concentration- and time-dependent manner with a half-maximum inhibitory concentration (IC_50_) of 78.4 nM for currents elicited at +40 mV (Fig. 1b–d). The AC-1 compound exhibits remarkable specificity for the Kv7.1/KCNE1 complex. Co-expression of Kv7.1 with other KCNE isoforms (KCNE2-5) generates currents that are insensitive to AC-1 (Fig. 1e). Moreover, AC-1 does not affect homomeric Kv7.1, Kv7.2 or Kv1.5 channels (Fig. 1e,f). To quantify the effects of KCNE1 on the sensitivity of Kv7.1/KCNE1 channels to AC-1, different amounts of KCNE1 cRNA (0.005–5 ng oocyte^−1^) were co-injected with a fixed amount of Kv7.1 cRNA (5 ng oocyte^−1^). Increasing the ratio of KCNE1:Kv7.1 cRNA increases current amplitude, slows its rate of activation and causes an apparent rightward shift in the voltage dependence of channel activation (Supplementary Fig. 1). Low KCNE1 levels leads to channels with low AC-1 sensitivity, whereas high KCNE1:Kv7.1 ratios induce currents that are strongly inhibited by AC-1 (Fig. 1g,h).

### AC-1 modifies gating of Kv7.1/KCNE1 channels

**Figure 2:**
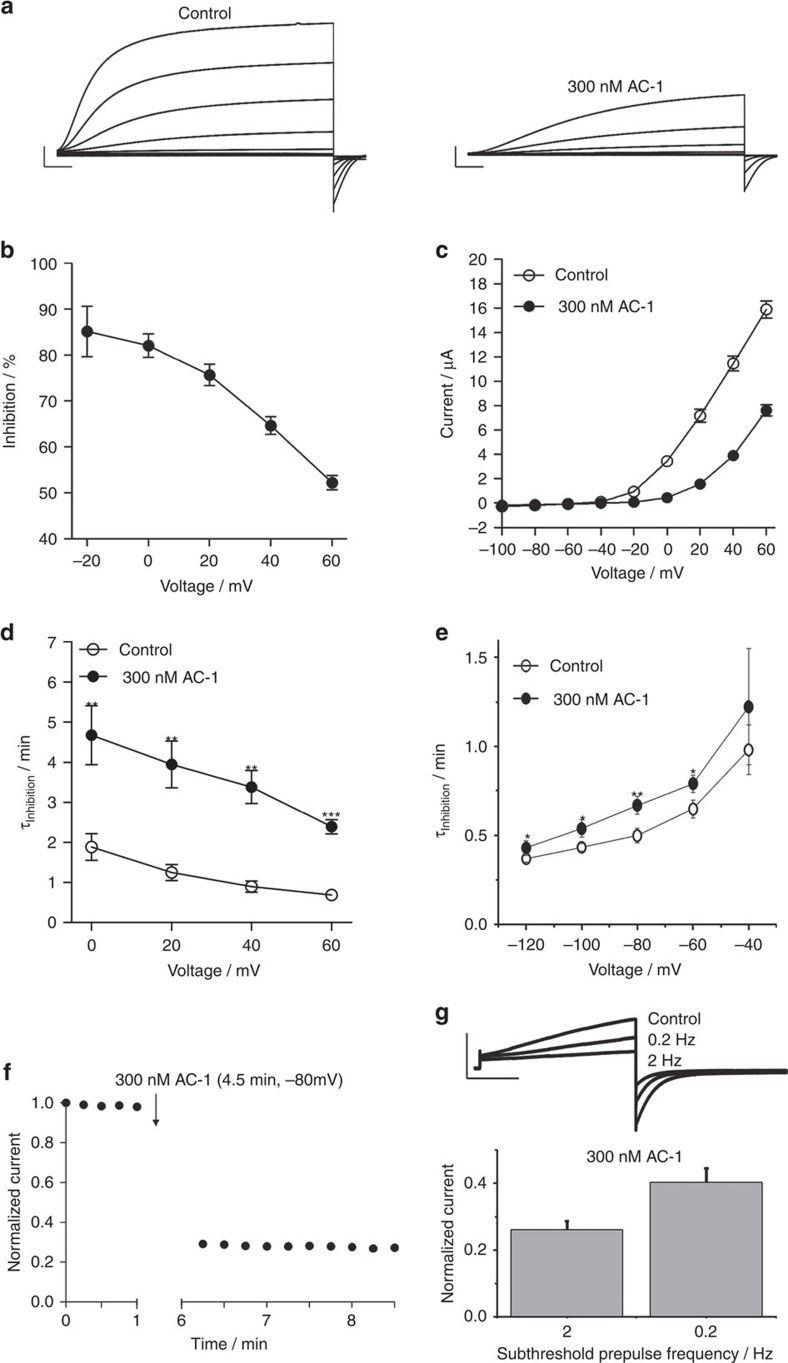
AC-1 alters activation kinetics and shifts voltage-dependence of activation. (**a**) Example of Kv7.1/KCNE1 current traces recorded before and after application of 300 nM AC-1. Currents were elicited with 10 s pulses to potentials of −100 mV to +60 mV, applied in 20 mV increments from a holding potential of −80 mV. Tail currents were recorded at −120 mV (scale bars indicate 2 μA, 1 s). (**b**) Voltage dependence of AC-1 inhibition determined as percent change of current amplitude after application of 300 nM AC-1 (*n*=15,±s.e.m.). (**c**) Rates of current activation are slowed in the presence of 300 nM AC-1. Activation was described using a single exponential function. *τ*_activation_ was plotted against the test potential (*n*=5, ±s.e.m.; Student's *t*-test; **P<0.01, ***P<0.001). (**d**) AC-1 shifts the voltage dependence of channel activation to more positive potentials. Macoscopic currents analysed at the end of test pulses and plotted against their respective test potential. (*n*=15, ±s.e.m.; Student's *t*-test). (**e**) Currents were activated by a 5 s depolarizing pulse to −20 mV. Tail currents were recorded at different test potentials ranging from −140 mV to −40 mV, applied in 20 mV increments. 300 nM AC-1 decreases the rate of current deactivation measured by fitting traces to a single exponential function to determine *τ*_deactivation_ (*n*=7–9, ±s.e.m.; Student's *t*-test). (**f**) Channels were held in closed state during application of 300 nM AC-1 by clamping the oocyte to −80 mV for 4.5 min without pulsing. On re-initiation of pulsing to +40 mV, the inhibition of current magnitude was fully developed, indicating that AC-1 is able to access its binding site when channels are in the closed state. (**g**) Channels were activated by 5 sec 0 mV pulses to obtain a control value. Subsequently, 300 nM AC-1 were applied and channels were preconditioned for 1 min by 300 msec −40 mV -subthreshold prepulsing at 2 and 0.2 Hz (scale bars indicate 1 μA, 1 s). At the end of preconditioning channels were activated again by 5 s 0 mV pulses and the current amplitudes at the end of the activating pulse were normalized to the control value (*n*=8, ±s.e.m.; Student's *t*-test).

At sufficiently high concentrations, pore blockers of ion channels can completely inhibit ion flux. In contrast, the maximum reduction of Kv7.1/KCNE1 channel currents by AC-1 was about 90%, even at concentrations ∼100-fold greater than the IC_50_ (Fig. 1b,c,h). Although not definitive, this observation was an initial indication that AC-1 may act as a gating modifier. Inhibition of Kv7.1/KCNE1 channels by AC-1 was voltage dependent, with the relief of inhibition apparent at higher membrane potentials (Fig. 2a,b). Accurate estimates of conductance–voltage relationships are challenging for I_Ks_ (Kv7.1/KCNE1) currents due to contamination by endogenous currents that are activated by long pulses at high potentials. However, significantly higher voltages (∼20–40 mV) are required to elicit measurable channel opening in the presence of AC-1 (Fig. 2c), indicating a considerable depolarizing shift in the voltage-dependence of activation. We also observed a strong deceleration of channel activation, but little to no effect on channel deactivation kinetics (depending on whether channels were activated with prepulses to −20 mV versus 40 mV) in the presence of AC-1 (Fig. 2a,d,e, Supplementary Fig. 2). These data are consistent with a depolarizing shift in the voltage-dependence of activation, and suggest that AC-1 likely acts by stabilizing a closed channel state. Some important distinctions should be noted between different mechanisms of gating modifiers. Hanatoxin, a gating modifier of Kv2.1 channels^32^, causes pronounced acceleration of channel deactivation, because it remains bound to channels throughout the gating process and destabilizes the open state. A very different mechanism has been proposed for gambierol, a gating modifier of Kv3.1 channels, which binds to closed channels and must dissociate in order for channels to open^33^. Effects of AC-1 may be more consistent with this 2nd model, particularly the very minor effect on channel deactivation which suggests that AC-1 may not remain associated with open channels.

To directly test whether AC-1 preferentially accesses and stabilizes a non-conducting closed state, we applied the compound to oocytes clamped at −80 mV and analysed current inhibition by resuming pulsing after 4.5 min. Current was highly inhibited during the first post-rest pulse, suggesting that AC-1 can access its binding site when the channel is in a closed state (Fig. 2f). Conditioning subthreshold prepulses at 0.2 and 2 Hz also revealed use-dependent drug effects suggesting inhibition in preopen states (Fig. 2g). We also tested the onset of current inhibition during wash-in of AC-1 while holding oocytes at various membrane potentials. The onset of block was significantly faster with the more negative holding potentials, suggesting that the drug interacts preferentially with a closed/pre-open channel state (Supplementary Fig. 3).

### Ala scanning of S4, S5, S6 and proximal C terminus of Kv7.1

**Figure 3:**
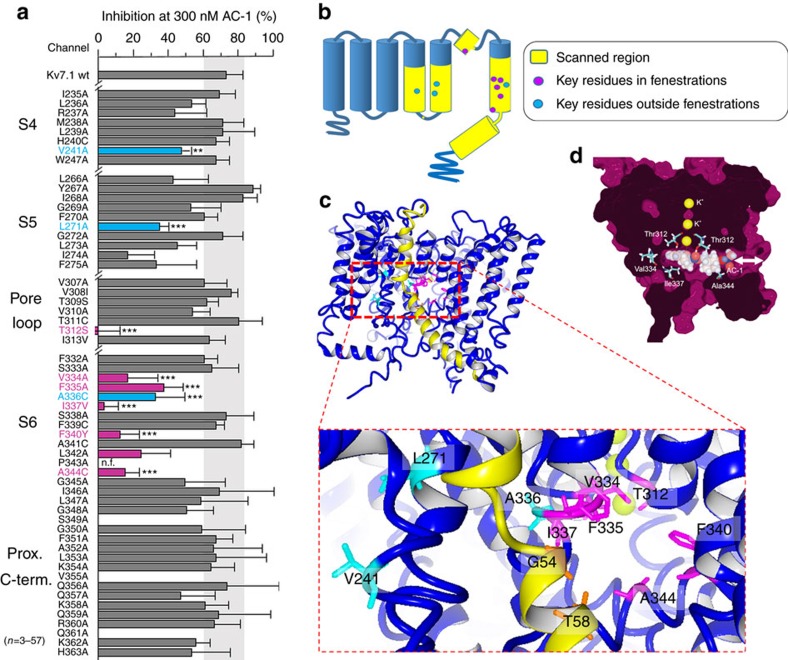
Putative binding mode of AC-1. (**a**) Inhibition of wt and mutant Kv7.1/KCNE1 channels by 300 nM AC-1. Influence of amino acid exchange (yellow) on channel sensitivity to 300 nM AC-1 was investigated using alanine scanning combined with TEVC. Inhibition was determined as percent change in current amplitude at the end of a depolarizing test pulse (*n*=4–57, ±s.e.m.; one way analysis of variance, Dunnett's *post hoc* test; ***P<0.001). (**b**) Cartoon of a single Kv7.1 channel subunit with scanned region in yellow. The circles indicate positions of mutations that significantly alter AC-1 sensitivity (key residues). Magenta filled circles indicate residues that face into the fenestration and blue filled circles mark residues that do not face fenestrations. (**c**) A preopen closed state model of Kv7.1/KCNE1 (Kv7.1 colored blue, KCNE1 colored yellow) with key residues highlighted in magenta (fenestration facing), blue non-fenestration facing and orange (fenestration facing in KCNE1). (**d**) Molecular docking of AC-1 to a homology model of Kv7.1/KCNE1 (ref. 33). AC-1 was positioned in close proximity to the amino acid residues identified by alanine scanning. AC-1 is located in a fenestration, which is only formed in the presence of KCNE1 (ref. 35).

Ala scanning was previously used to identify specific residues that form the putative binding sites for chromanol and benzodiazepine derivatives that are considered to be classical pore blockers of Kv7.1 channels^20^,^21^. Here we applied Ala scanning to multiple segments of the Kv7.1 α-subunit to identify potential AC-1 interaction sites in the extended pore region of Kv7.1/KCNE1 channels (Fig. 3a). In cases where mutation to Ala precluded channel function, alternative residues were introduced. This approach identified one residue in S4, two in S5, one residue at the base of the pore helix, and seven residues in S6, that markedly reduced AC-1 sensitivity when mutated (Fig. 3b, Supplementary Fig. 4, Table 1). The S6 residues identified by Ala scanning form a cluster in the channel structure, but do not all face the central cavity in three-dimensional (3D) models of open, closed^34^ or preopen states^35^. Instead, most of these crucial residues cluster around a ‘window' formed uniquely in the preopen–closed state model of the Kv7.1/KCNE1 channel^33^ (Fig. 3c, magenta residues). This window structure resembles the fenestrations described for the NavAb channel and proposed to be a binding site for local anesthetics that inhibit Nav channels^36^,^37^. Docking of AC-1 to our previously published preopen–closed state model^35^ and subsequent MD-simulation was performed and indicated that AC-1 was stable in two positions along the longitudinal axis of the fenestrations (Fig. 3d). By contrast, *in silico* models of the closed and open states do not exhibit clear fenestrations (Supplementary Fig. 5) and thus, AC-1 cannot interact with this cavity in these channel states.

### Chemical modification of the AC-1 scaffold

**Figure 4:**
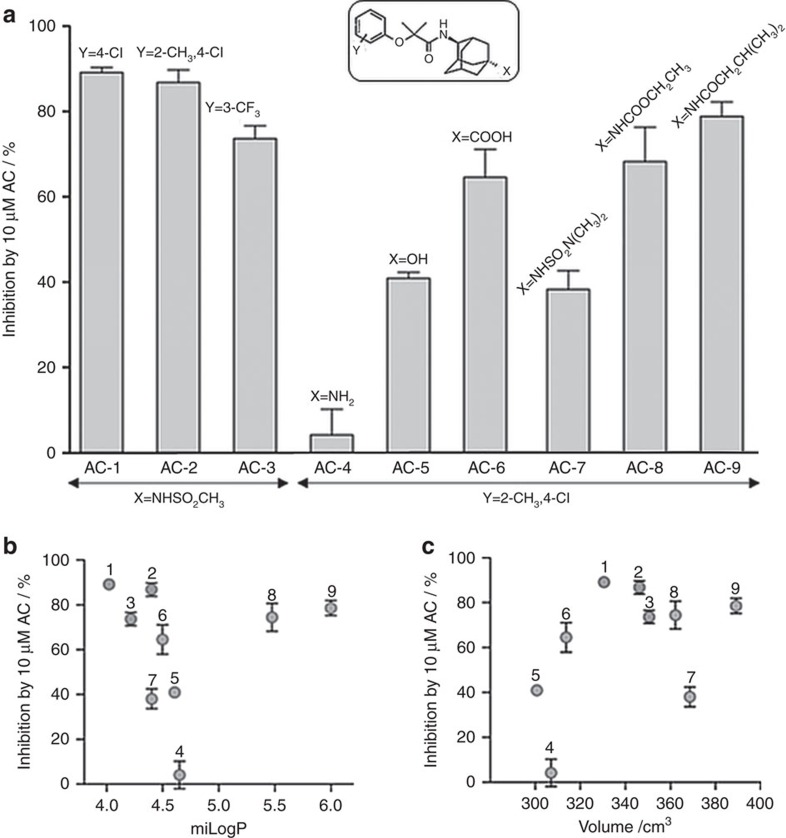
Structure activity relationship of adamantane compounds. (**a**) Inhibitory effect of different AC derivatives (10 μM) on Kv7.1/KCNE1 channels. Chemical modifications at both ends of AC-1 were investigated as indicated. Inhibition was determined as percent change in current amplitude at the end of a depolarizing test pulse to +40 mV (*n*=3–5, ±s.e.m.). (**b**) Lack of correlation between inhibitory effect and hydrophobicity of AC compounds (*n*=3–5, ±s.e.m.). (**c**) Lack of correlation between inhibitory effect and volume of AC derivatives (*n*=3–5, ±s.e.m.). miLog *P* values and volume were calculated using Property Calculator (Molinspiration Cheminformatics).

We also investigated the functional effects of a panel of AC-1 analogs^30^ with chemical modifications at either end of the drug scaffold (Supplementary Table 1). Variation at position Y had little effect on inhibition. Introduction of substituents with variable volume and hydrophobicity at position X showed that small polar substituents reduce inhibition, whereas bulky substituents are tolerated and can increase apparent affinity (Fig. 4a). The potency of ACs was not dependent on hydrophobicity, quantified as logP using the Molinspiration logP calculator (Fig. 4b) or volume (Fig. 4c). Notably, results obtained with AC-9, an isobutyramide analog of the methylsulfonamide AC-2, indicate that the sulfonamide can be replaced with a carboxamide, and that relatively large lipophilic groups can be accommodated. These data clearly show that the AC-1 scaffold can be significantly expanded at either longitudinal end, suggesting that the AC binding site is not precisely length-delimited. We speculate that this SAR argues against the notion of a central cavity-constrained binding site, which would limit the dimensions by which drugs could be expanded while retaining effectiveness.

### Photoaffinity labelling approach to identify AC interactions

**Figure 5:**
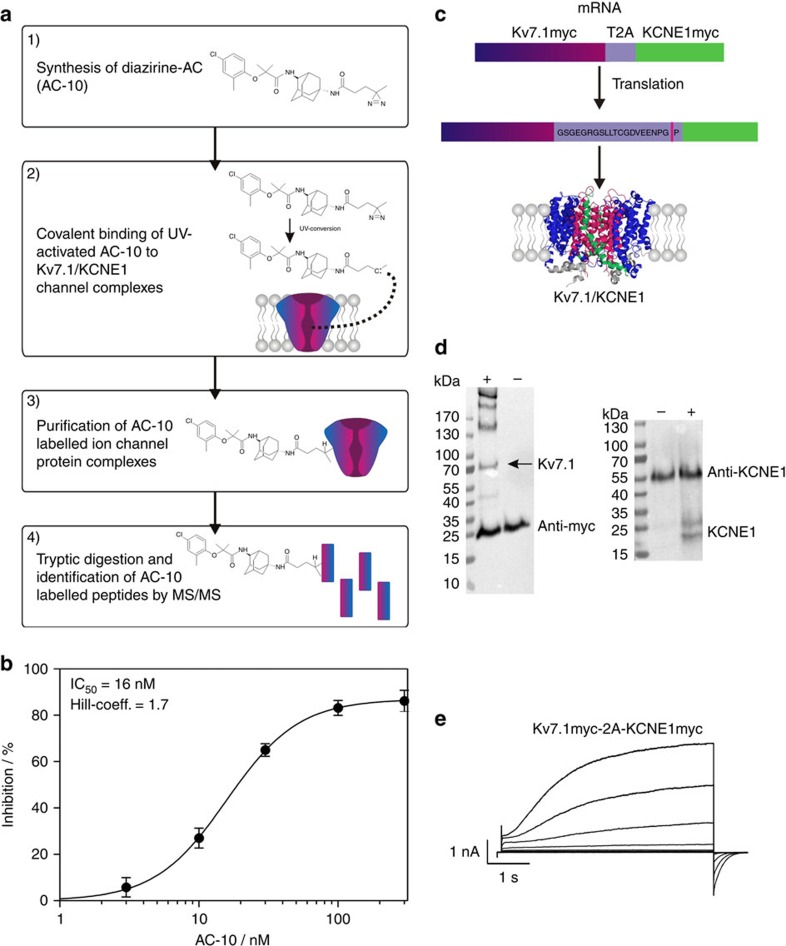
PAL-based approach to identify AC binding site. (**a**) Schematic view of the PAL-based approach to investigate the binding site of AC-1. (**b**) Concentration-response curve for AC-10, the UV-active diazirine derivate of AC-1. The inhibitory effect of AC-10 was determined in CHO cells stably expressing Kv7.1/KCNE1. Inhibition was determined as percent change in current amplitude at the end of the depolarizing test pulse to +40 mV (±s.e.m.). (**c**) A new cDNA-construct (Kv7.1myc-2A-KCNE1myc in *pXOOM*) allows for functional expression of myc-tagged Kv7.1 and KCNE1 in HEK293T cells. In this construct, cDNAs of Kv7.1 and KCNE1 are linked via the T2A peptide sequence, which mediates co-translational protein cleavage^42^. (**d**) Western blot analysis of affinity purified Kv7.1 and KCNE1 proteins. Kv7.1myc was purified using myc-agarose beads KCNE1 was purified using anti-KCNE1 coupled protein-A-sepharose beads (right). In both western blots, ‘+' indicates transfected cells, ‘−' indicates non-transfected control cells. Antibodies used for affinity purification are detected in ‘+' and ‘−'. Additional protein bands proved the expression of Kv7.1 and KCNE1 proteins. It should be noted, that KCNE1, as a myc-tagged protein, should also be purified using the myc-agarose beads. Since the respective KCNE1 band is of comparable size to the anti-myc band, it is not possible to distinguish between them. Thus, KCNE1 expression was instead proven using the KCNE1 antibody. (**e**) Functional expression of Kv7.1myc-2A-KCNE1myc in HEK293T cells (scale bars indicate 1 nA, 1 s).

Interpretation of mutagenesis-based investigation of drug binding sites is often hampered by the possibility of secondary allosteric effects that impact drug binding or alter drug response with no change in binding affinity. Therefore, we complemented our mutagenesis and modelling findings by developing a photoaffinity labelling (PAL)-based approach to directly identify regions of the Kv7.1/KCNE1 complex that interact with the AC compounds. We designed and synthesized an AC-9 analog with a photo-activatable cross-linking moiety that could covalently bind to the Kv7.1/KCNE1 channel complex (Fig. 5a, step 1–2). Labelled channel complexes were purified, and modified peptides were identified using MS/MS spectrometry (Fig. 5a, step 3–4). The diazirine substituted AC analog used for chemical cross linking was synthesized by coupling an NHS-diazirine to the amino group of AC-4 (Fig. 4) to generate AC-10 (Supplementary Fig. 6).

**Figure 6:**
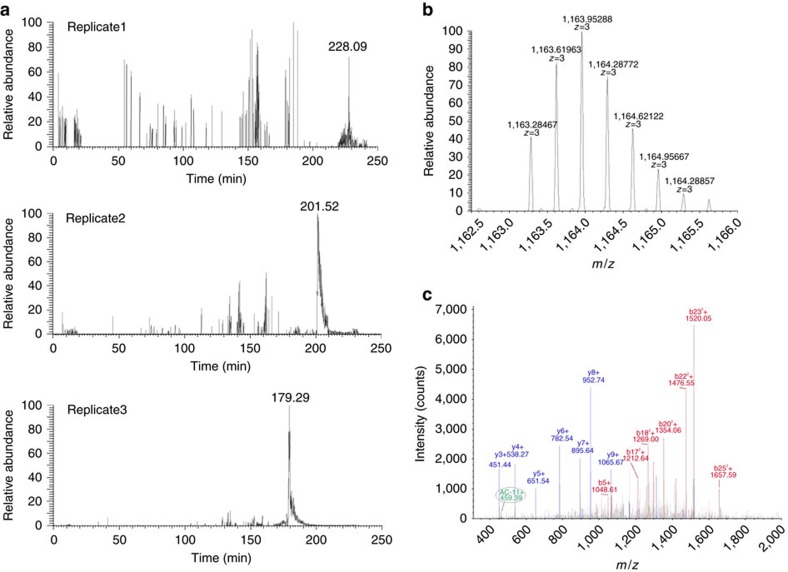
MS/MS-analysis. (**a**) Extracted ion chromatograms of the triply charged species of the KCNE1 peptide ^42^LEALYVLMVLGFFGFFTLGIMLSYIR^67^ with AC-10 modification (1163.28 m/z) identified across three replicate LC-MS/MS analyses. Isotope pattern of the [M+3H]^3+^ precursor of AC-10 modified peptide ^42^LEALYVLMVLGFFGFFTLGIMLSYIR^67^ (**b**) and corresponding MS/MS spectra with b- (red) and y-ion annotation (blue) (**c**). The characteristic marker ion of AC-10 (459.39*m/z*) is observed in the MS/MS spectrum. The consecutive y-ion series y_2_–y_9_ of respective amino acids is nicely displayed.

Diazirines can be activated by UV irradiation at 350 nm, and generate highly reactive carbene species (Supplementary Fig. 6) leading to covalent binding to all natural amino acids^38^,^39^. AC-10 inhibited Kv7.1/KCNE1 channels with an IC_50_ of 16 nM, indicating that the diazirine adduct does not weaken drug binding. AC-10 did not inhibit homomeric Kv7.1 channels (Fig. 5b, Supplementary Fig. 6c), Due to its very high apparent affinity, the effects of non UV-activated AC-10 could only be partially reversed by washout (40% inhibition after 38 min washout). After UV irradiation, current inhibition by AC-10 was mostly irreversible, suggesting that AC-10 was covalently bound and thus locked the channel in a non-conductive state (75% inhibition after 38 min washout, supplementary Fig. 6d). To achieve equal overexpression of subunits and to facilitate subsequent protein isolation, a bicistronic vector containing the cDNAs of c-myc tagged Kv7.1 and KCNE1 were linked via a T2A peptide sequence (Supplementary Fig. 7, Fig. 5c), a ∼20 amino acid sequence which mediates co-translational cleavage of polyproteins^40^,^41^,^42^. Transfection of HEK293T cells with this bicistronic vector leads to expression of Kv7.1myc and KCNE1myc proteins that were isolated via affinity chromatography and detected by Western blot analysis using a c-myc or anti-KCNE1 antibody (Fig. 5d). Functional expression of Kv7.1/KCNE1 channels in HEK293T cells was verified by patch clamp experiments (Fig. 5e). AC-10 was applied and covalently bound to channels overexpressed in HEK293T cells by UV irradiation. The tagged channel proteins were then purified on anti-myc columns, digested by trypsin, and the AC-10 modified peptides detected by MS/MS spectrometry (Fig. 6a,b, Supplementary Fig. 8 shows coverage of MS/MS). The only AC-10-modified peptide identified corresponds to the transmembrane helix of the β-subunit KCNE1 (^42^LEALYVLMVLGFFGFFTLGIMLSYIR^67^, Fig. 6c). Due to limited resolution, it was not possible to explicitly identify a single AC-10-bound amino acid residue. Another consideration is that some flexibility of the diazirine moiety may enable cross-linking at multiple positions in the identified peptide region. Nevertheless, no other labelled peptides (from Kv7.1 or KCNE1) were detected in three experimental trials, suggesting that there is no promiscuous cross-linking of the diazirine compound throughout the channel. Furthermore, when KCNE1 was expressed alone or in complex with Kv2.1, no KCNE1 modification was detected, indicating that both Kv7.1 and KCNE1 are required for AC-10 modification. Spectra for KCNE1 alone or in complex with Kv2.1 do not provide any evidence for modification by AC-10 (Supplementary Fig. 9). Rather, cross-linking with KCNE1 is very specific and reflects cross-linking taking place in a narrowly defined binding orientation that requires co-assembly with Kv7.1.

### Cys scanning of the KCNE1 transmembrane segment

**Figure 7:**
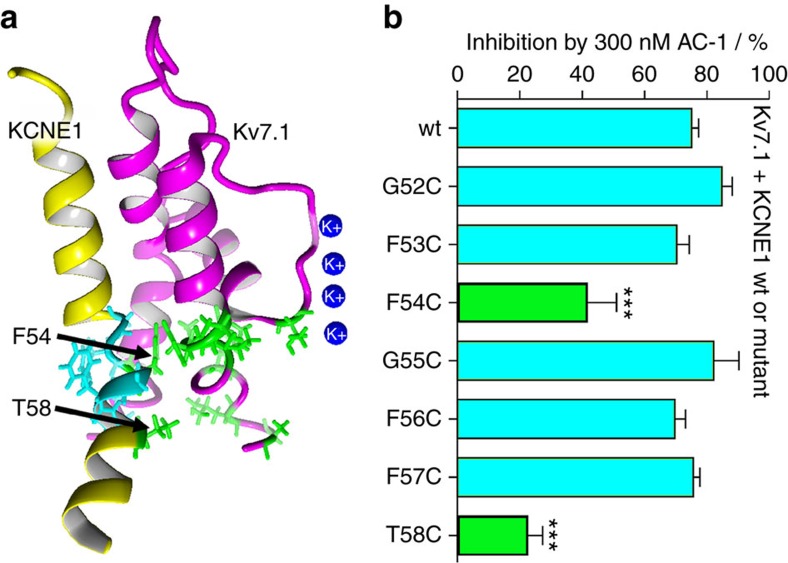
Single point mutagenesis of the transmembrane region of KCNE1. (**a**) Location of the transmembrane region of KCNE1 (KCNE1-TM) in a Kv7.1/KCNE1 channel complex. (**b**) Inhibition of Kv7.1/KCNE1 channel complexes by 300 nM AC-1. Single point mutations were introduced as indicated and inhibition was determined as percent change in current amplitude at the end of a depolarizing test pulse to +40 mV (*n*=3–5, ±s.e.m.; one-way analysis of variance, Dunnet's *post hoc* test; ***P<0.001).

The β-subunit KCNE1 markedly increases sensitivity of Kv7.1 channels to classical pore blockers such as chromanol 293B and the benzodiazepine L-7 (refs 20, 21, 29). KCNE1 does not directly participate in binding of these inhibitors, but is thought to facilitate ligand binding to the α-subunit Kv7.1 via an allosteric mechanism^29^. In contrast, our PAL-based experiments indicate that the transmembrane segment of KCNE1 may be directly involved in binding of AC-1/AC-10. To further investigate whether specific KCNE1 residues interact with AC-1, cysteine substitutions of residues located in the central transmembrane region of KCNE1 (residues 52–58) were generated. Based on response of the mutant channels to 0.3 μM AC-1, Phe54 and Thr58 were identified as crucial residues for AC-1 binding (Fig. 7).

### Constrained AC-1 docking

**Figure 8:**
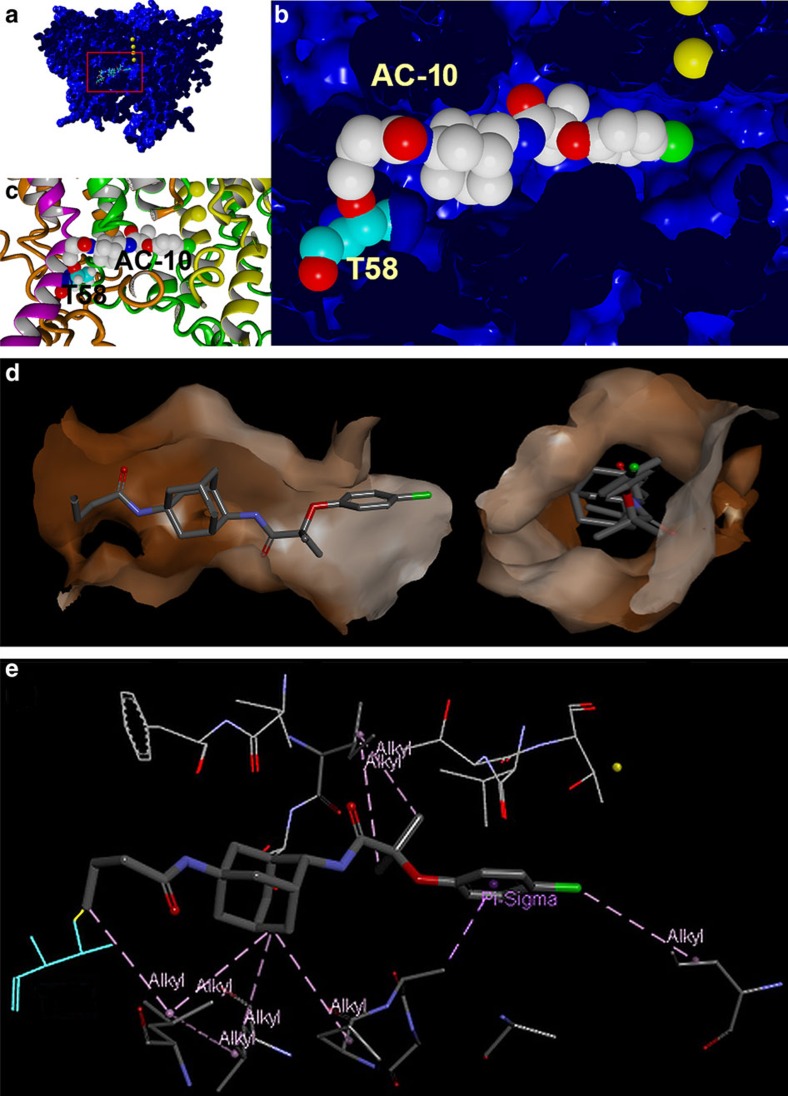
Constrained docking of AC-10 to Kv7.1 channel. (**a**) AC-10 carbene docked by constraining the distance (2 ±1 Å) to the KCNE1 Thr58 side chain. The energy minimized model complex is shown. Kv7.1/KCNE1 was surface rendered, cut in the middle and colored blue, KCNE1 Thr58 is presented in ball-representation and CPK color coding with carbons in cyan. Compound AC-10 is shown in ball-representation and standard CPK color coding. K^+^-ions are shown as yellow spheres. (**b**) Close-up of AC-10 in its binding pocket. (**c**) Close-up representation of similar region, but with protein shown as ribbons (KCNE1 in magenta, Kv7.1 subunits in yellow green orange). (**d**) The AC binding site is formed by several residues that surround a tunnel-like structure (fenestration) that bridges the inner cavity (left) and the membrane (right). The surface lining the fenestration is hydrophobic, especially in the region around the adamantine of AC-1. (**e**) The specific ligand-receptor interactions determined using Discovery Studio 4.0. are: six alkyl interactions (pink), one alkyl-pi interaction (pink) and one carbon interaction (green).

Results from PAL experiments, along with mutational scans of Kv7.1 and KCNE1, were used to facilitate a spatially restrained modelling of AC interaction with the Kv7.1/KCNE1 complex. To generate the model, AC-10 was introduced by swapping/adding atoms in the AC-1–Kv7.1/KCNE1 model shown in Fig. 3c. Next, the distance between the reactive AC-10 group and the side chain of Thr58 was constrained to a distance that allowed for covalent binding. The resulting model shows AC-10 bound in a position within the fenestrations of Kv7.1 (Fig. 8). We also performed 25 nsec simulations (all atoms mobile) on the AC-10–Kv7.1-KCNE1 model, and observed very stable binding in this conformation throughout the simulation. A parallel simulation on the identical Kv7.1-KCNE1 model without AC-10 showed significant fluctuation throughout the channel complex, indicating that AC-10 may stabilize the channel complex (Supplementary Fig. 10).

## Discussion

**Figure 9:**
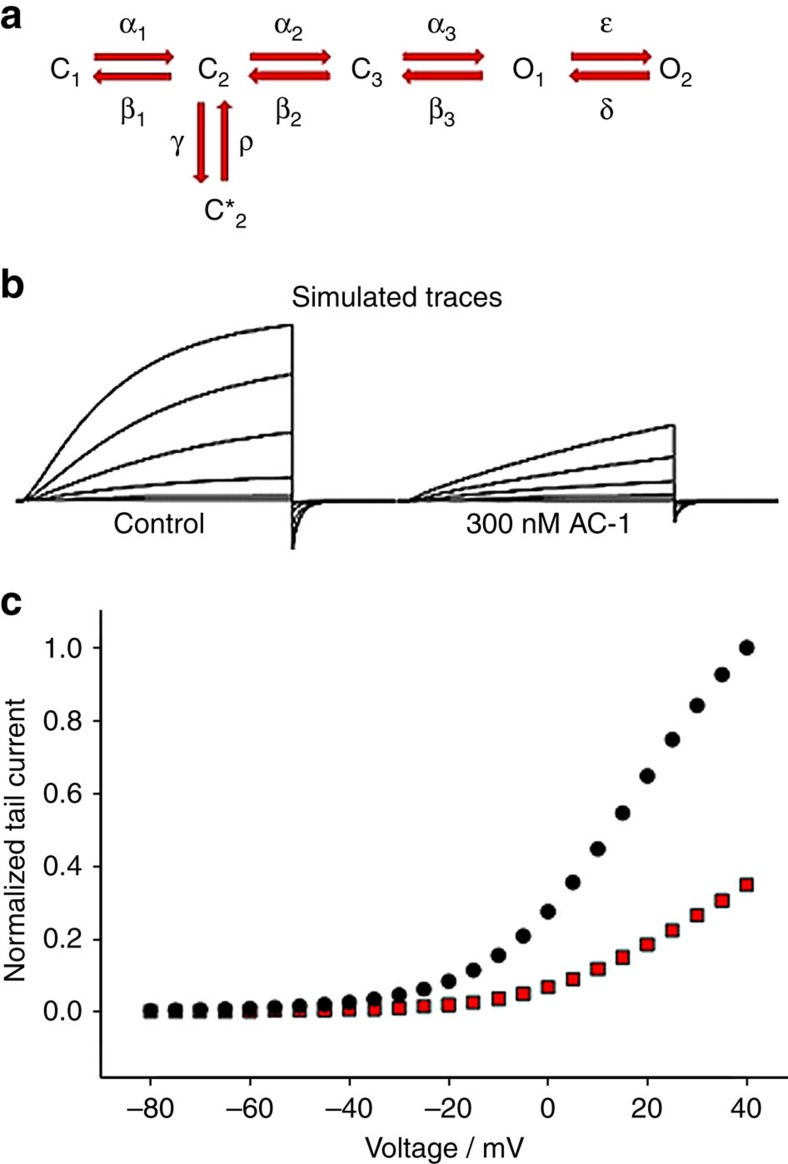
Markov state modelling of AC-1 inhibition. Markov state modelling was used to simulate current traces under control and 300 nM AC-1. The state model as indicated in **a** was used. The rate constants (in s^−1^) had the following voltage dependence (*φ*=VF/RT) (where V is the membrane voltage, F Faraday's constant, R the gas constant and T absolute temperature): *α*_1_=exp(0.47 φ); *β*_1_=0.2 × exp(−0.35 φ); *α*_2_=0.46 × exp(0.47 φ); *β*_2_=3.3 × exp(−0.35 φ); *α*_3_=24 × exp(0.06 φ); *β*_3_=19 × exp(−0.007 φ); *ɛ*=4.6 × exp(0.8 φ); *δ*=1.4 × exp(−0.7 φ); *γ*=10. For unbound channels ρ=10, and for AC-1 bound channels ρ=0.1. The voltage protocol used to simulate the traces shown in the figure was: holding potential −80 mV, steps from −40 to +60 mV in 20 mV steps for 5 s, tail potential −120 mV. Simulated traces are shown in (**b**). Simulated normalized peak inward tail currents at −120 mV are plotted as a function of the prepulse potential (5 s pulses, control in black and AC-1 in red circles **c**).

Potent inhibition of Kv7.1 channels by ACs requires the presence of KCNE1 β-subunits. KCNE1 subunits modify the gating of Kv7.1 and structural modelling suggests that these β-subunits also induce a structural change in the channel complex that provides a closed state-preferred binding site for the ACs. Several findings suggest that ACs inhibit current by stabilizing a non-conducting closed state rather than by occluding the ion-conducting pore. First, at saturating concentrations, AC-1 causes incomplete inhibition of current unlike the complete inhibition normally observed with a pore blocker. Second, reduced channel inhibition at higher voltages is associated with slowed activation kinetics, and a shifted voltage dependence of activation to more depolarized potentials. Third, inhibition by AC-1 is enhanced when channels are in a closed state (Fig. 2). Gating and AC-1 effects on Kv7.1/KCNE1 channel complexes can be described by a simple scheme (Fig. 9a). In this model, C_1_, C_2_, C_3_ are closed states, O_1_, O_2_ are open states and C_2_* represents a stable (‘inactivated') closed state. The kinetic behavior of AC-1 is consistent with drug binding leading to stabilization of C_2_* (refs 19, 33). In our kinetic model, binding of AC-1 is presumed to decrease the rate-constant from C2* to C2 by 100-fold, largely recapitulating the kinetic effects of the drug (Fig. 9b). An inference from this model is that AC-1 may dissociate from the channel during this C2*→C2 transition, thereby delaying channel activation with little or no effect on deactivation kinetics (Fig. 2). The model also recapitulates a shift of the voltage dependence of activation (Fig. 9c). Other kinetic models in which AC-1 stabilizes more deeply closed states are also compatible with the data, however, stabilization of the preopen closed state model is additionally suggested by *in silico* MD simulation, and experimental data including a faster onset of block with mild depolarization, and use-dependent inhibition with modest depolarizations (Supplementary Fig. 9, Fig. 2g). Classical inhibitors of Kv7.1 channels such as chromanol 293B and the benzodiazepine L-7 disrupt ion permeation by interacting with the pore-lining S6 segment and the lower pore helix/selectivity filter of the Kv7.1 α-subunit^20^,^21^,^28^. In contrast to this classic view of pore plugging by a blocker, quinidine allosterically inhibits Kv7.1 channels by binding to a small lateral pocket formed by the S4-S5 linker and S6 (ref. 26). Importantly, all three of these compounds inhibit both homomeric Kv7.1 and heteromeric Kv7.1/KCNE1 channels.

In this study, we identified the binding mode for a new class of specific inhibitors of Kv7.1/KCNE1 channels. We propose that the β-subunit KCNE1 induces the formation of fenestrations in the pore domain of Kv7.1 that are required for binding of AC derivatives. For commonly used inhibitors of Kv7.1 (for example, chromanol 293B), it has been reported that KCNE1 increases drug sensitivity of the channel^20^,^21^ by an allosteric mechanism^29^, although there is not the same stringent requirement for KCNE1 as we observe for the AC compounds. The formation of lateral fenestrations was seen in a structural model of Kv7.1/KCNE1 representing a preopen-closed state, with the S4 voltage sensors in the up-state and the pore gate (S6-bundle crossing) in a closed state configuration, but no fenestrations were observed in the open channel model^33^. A similar fenestration was described in the crystal structure of NavAb, a voltage-gated sodium channel from *Arcobacter butzler*^34^,^35^, and have been proposed to provide a pathway into the central cavity for local anesthetics. The side pockets described in Kv1.5 channels are smaller than Na_V_-fenestrations and appear to permit selective targeting of these channels by Psora-4 (ref. 25). Our scanning mutagenesis experiments identified many residues in the fenestration that are important for AC-1 activity. In addition, residues in S4 (Val241), S5 (Leu271), the base of the pore helix (Thr312), and at the S6 bundle crossing (Ala344) were also identified by this approach. We suspect that the effects of more distant S4 and S5 residues may be allosteric, as we did not observe any indication of drug binding in these regions using the PAL approach. Moreover, the selectivity filter/pore helix structure is allosterically linked to the activation gate (the S6-bundle crossing), along with S4 and S5 (ref. 41). This hypothesis is supported by our *in silico* observation that AC-10 binding causes structural stabilization (reduced root mean square fluctuation (RMSF)) of the whole Kv7.1/KCNE1 (transmembrane helix) channel complex, indicative of allosteric coupling of drug binding to other channel segments (Supplementary Fig. 9).

Chemical substitutions at either end of the AC scaffold suggest some spatial freedom with regard to potency for inhibition of Kv7.1/KCNE1 channels. This finding is consistent with a horizontal position of AC-1 relative to the pore axis (Fig. 3c). However, based on these experiments it was not possible to determine exactly how far ACs are positioned towards the central cavity or the outer face of the pore domain. The cross linking of AC-10 to the KCNE1 transmembrane α-helix suggests that ACs may bind within the fenestrations, thus stabilizing the channel complexes in a preopen-closed state similar to the 3D model of the Kv7.1/KCNE1 complex proposed in 2011 (ref. 33). KCNE1 Thr58 and Phe54 are located immediately adjacent to the outer fenestration in Kv7.1, consistent with spatial continuity of all the residues identified as important by mutagenesis and, were used to guide a constrained-docking of AC-10 to the Kv7.1/KCNE1 model. AC-10 was modelled to be in a slightly more lateral position within the KCNE1-induced fenestrations in Kv7.1 compared with the initial docking of AC-1 (Figs 3c, 8). Covalent compound binding on ultraviolet radiation allowed for a physical/chemical determination of binding parameters that cannot be achieved solely by the classical mutagenesis scanning approach. Even though no evidence was found in this study, the existence of further lower affinity binding sites in the channel complex cannot be excluded.

The KCNE1 β-subunit induced fenestration and subsequent specific inhibition of hetero-multimeric channels described here for Kv7.1/KCNE1 could also be present in other voltage-gated channels that interact with other KCNE β-subunits. As several channels have been reported to interact with one or more of the five KCNE proteins, we suggest that β-subunit-induced fenestrations may occur in other channels, leading to formation of inhibitor binding sites similar to the one described herein for ACs. In the case of Kv7.1, only KCNE1 has been shown to enable the formation of a highly specific binding site (Fig. 1). Tissue-specific targeting of channels may potentially be achieved by generation of inhibitors acting on channels with highly constrained combinations of Kv and KCNE subunits. In this context, inhibitors with hydrophobic structure similar to adamantane represent a promising new pharmacophore for development of new, highly selective channel inhibitors.

## Methods

### Chemicals

AC derivates were synthesized at Janssen Pharmaceutica. Syntheses were described in a patent by Jaroskova *et al*.^30^. AC-10 was synthetized from condensation of AC4 with SDA (NHS-Diazirine) as shown in supplementary Fig. 7. N-[(1 R,3 S)-5-amino-2-adamantyl]-2-(4-chloro-2-methyl-phenoxy)-2-methyl-propanamide (AC4, 0.050 g, 0.13 mmol) and 3H-diazirine-3-propanoic acid, 3-methyl-, 2,5-dioxo-1-pyrrolidinyl ester (SDA, NHS-Diazirine, Pierce 26167, 0.025 g, 0.11 mmol) were dissolved in dichloromethane (2 ml), triethylamine (0.2 ml, 1.44 mmol) was added, and the mixture was stirred for 48 h at room temperature. The reaction mixture was diluted with dichloromethane (10 ml), washed with 1M HCl (2 × 5 ml), 1M NaOH (2 × 5 ml), and saturated brine solution (2 × 5 ml), dried (MgSO_4_), and evaporated till dryness. The residue was crystallized from diisoproyl ether, yielding pure AC10 (2-(4-chloro-2-methyl-phenoxy)-2-methyl-N-[(1 R,3 S)-5-[3-(3-methyldiazirin-3-yl)propanoylamino]-2-adamantyl]propanamide, 27.5 mg, 0.056 mmol, 43% yield). ^1^H NMR (400 MHz, DMSO-d6) 0.91 (s, 3H, C(N_2_)-CH_3_), 1.35 (bd, 2H), 1.45 (s, 6H, 2 × CH_3_), 1.50, (t, 2H, *J*=7 Hz, -CH_2_-CH_2_), 2.07 (bd, 2H), 1.68-2.05 (m, 11H), 2.24 (s, 3H, Ar-CH_3_), 3.71 (bd, 1H, *J*=5H, CONHCH), 6.75 (1H, d, *J*=7 Hz, Ar-H), 7.15 (dd, 1H, *J*=7 Hz, *J*=1 Hz, Ar-H), 7.25 (s, 1H, CNHCO),7.3 (d, *J*=5H, CONHCH,) 7.4 (d, *J*=1 Hz, Ar-H). LC/MS (ESI positive) *m/z* 487, 489.

### Molecular biology, cRNA synthesis

Template cDNAs (wildtype or mutant human Kv7.1 in pSGEM, wildtype human KCNE1 in pSP64) were linearized with a suitable restriction enzyme (Nhe I/pSGEM, Eco RI/pSP64). cRNA was synthesized from 1 μg linearized cDNA by *in vitro* transcription (mMessage mMachine T7 or SP6 kit, Ambion, Life Technologies Darmstadt, Germany). cRNA concentrations were determined by photometry (Nanodrop, Peqlab, Erlangen, Germany) and cRNA quality was checked by agarose gel electrophoresis.

### Oocyte isolation and injection

Oocytes were commercially obtained from EcoCyte Bioscience (Castrop-Rauxel, Germany). Injection of stage 4 or 5 oocytes was performed using a nanoliter injector 2000 (WPI, Berlin, Germany). For standard pharmacological measurements, each oocyte was injected with 5 ng Kv7.1 cRNA alone or with 1 ng KCNE1 cRNA. To investigate the influence of KCNE1 on AC-1 sensitivity of Kv7.1 tetramers, 5 ng Kv7.1 cRNA was injected with different amounts of KCNE1 cRNA (0 ng, 0.005 ng, 0.05 ng, 0.5 ng and 5 ng). Injected oocytes were maintained in an incubator at 17 °C for 4–5 days in standard Barth solution (EcoCyte Bioscience, Castrop-Rauxel, Germany) supplemented with 90 μg ml^−1^ theophylline, 63 μg ml^−1^ penicillin, 40 μg ml^−1^ streptomycin, and 100 μg ml^−1^ gentamycin until used for two electrode voltage clamp (TEVC) measurements.

### Two electrode voltage clamp

TEVC measurements were performed using a TurboTec-10CD amplifier (npi Electronic, Tamm, Germany) and an ITC-16 AD/DA-interface (Instrutech Corporation, Port Washington, New York, USA). Pulse and PulseFit software (HEKA, Lambrecht/Pfalz, Germany) was used for data acquisition and analysis. During TEVC recordings, oocytes were constantly perfused with control solution (ND96, containing in mM: 96 NaCl, 4 KCl, 1.8 CaCl_2_, 1 MgCl_2_, 5 mM HEPES, pH 7.6 supplemented 0.1–0.3% DMSO). AC solutions were freshly prepared in ND96 from a 10 mM DMSO stock solution. Glass capillaries were pulled to a fine point to produce current and voltage electrodes that were back-filled with 3 M KCl. When filled with this solution, electrode resistances ranged from 0.5–1.5 MΩ. Different voltage pulse protocols were used as follows: (1) To study the inhibitory effect of different AC derivatives, Kv7.1/KCNE1 currents were elicited by repetitive 7 s pulses to +40 mV applied every 30 s from a holding potential of −80 mV. (2) To analyse the voltage dependence of channel activation, currents were elicited by 10 s pulses to test voltages between −100 and +60 mV, applied in 20 mV increments from a holding potential of −80 mV. Tail currents were recorded at −120 mV. For control we always expressed KCNE1 alone along mutant channels and mutant currents were only taken into account when it reached at least 2.5- to 3-fold the size of the background *Xenopus*-Kv7.1/hKCNE1 currents.

### Data analysis

Electrophysiological data were analysed using PulseFit software (HEKA, Lambrecht/Pfalz, Germany) and GraphPad Prism (GraphPad Software, Inc., La Jolla, California, USA). Channel inhibition was determined as reduction of whole cell current amplitude at the end of a 7 s test pulse to +40 mV. The concentration of compound that caused half-maximal inhibition (IC_50_) were determined using the Hill equation: I_drug_/I_max_=1/(1+[C/IC_50_]^H^), where I_drug_ is the current amplitude in the presence of the drug, I_max_ is the current amplitude before drug application, C is the drug concentration and H is the Hill coefficient^1^,^21^,^35^.

Activation curves were obtained as previously described for Kv7.1 homotetramers^42^. Initial tail currents were normalized to maximal values and plotted against the respective test potential^43^. Normalized data were fitted to a Boltzmann equation:


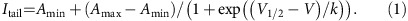


*V*_1/2_ is the voltage of half-maximal activation, k is the slope factor, *V* is the test voltage and *A*_max_ and *A*_min_ are the maximal and minimal tail current amplitudes. To analyse activation or deactivation kinetics, current traces were fitted to a single exponential function: *y*=*A*_0_+*A* × exp(−*t*/*τ*), where *A* is current amplitude, *A*_0_ is the steady-state amplitude and τ is the time constant for activation (*τ*_activation_) or deactivation (*τ*_deactivation_). A linear fit was performed using the linear function of the form *y*=*m* × log[AC−1]+b. Statistical analysis was performed using GraphPad prism. Student's *t*-test or a one-way analysis of variance followed by Dunnet's or Tukey's *post hoc* test were used. A *P* value <0.05 was considered statistically significant.

### Cell culture and transfection

HEK293T cells (DSMZ-No. ACC 635) were maintained in DMEM (Life Technologies, Darmstadt, Germany) supplemented with 10% fetal bovine serum (Life Technologies, Darmstadt, Germany) and 1 × non-essential amino acids (Sigma-Aldrich, Munich, Germany). For transfection, cells were seeded in 8 cm dishes and grown to 80–90% confluence. Cells were transfected with 12 μg plasmid cDNA (Kv7.1myc-2A-KCNE1myc in pXOOM) using Metafectene Pro (Biontex, Martinsried, Germany). Non-transfected cells served as controls.

### PAL with AC-10

For PAL of Kv7.1/KCNE1 channel complexes, transfected HEK293T cells were treated with photoactive AC-10. Non-transfected cells served as a control. In brief, cell culture medium was removed and cells were scrapped off the dish into 1 ml HBSS(−/−) (Life Technologies, Darmstadt, Germany). Cell suspensions of each condition were combined and centrifuged (309*g*, 5 min). Cells were resuspended in 10 ml ice-cold PBS (PBS in mM: 150 NaCl, 8 Na_2_HPO_4_, 2 KH_2_PO_4_), treated with 1 μM AC-10 and kept on ice in the dark for 30 min. After UV-radiation (350 nm) for 5 min, the cell suspension was centrifuged (309*g*, 5 min, 4 °C) and the packed cells were washed three times with 10 ml ice-cold PBS (309*g*, 5 min, 4 °C) to remove remaining AC-10. AC-10 labelled Kv7.1 and KCNE1 proteins were purified and analysed using western blot and immunodetection or MS/MS spectrometry.

### Cell lysis, affinity chromatography and western blot

For affinity purification of labelled Kv7.1 and KCNE1 proteins, transfected HEK293T cells were mechanically lysed. AC-10 treated or untreated cell suspensions were centrifuged (309*g*, 5 min, 4 °C) and pellets were resuspended in a five-fold volume of swelling buffer (containing in mM: 10 HEPES, 10 KCl, 1.5 MgCl_2_; pH 7.6) supplemented with protease inhibitor (Sigma-Aldrich, Munich, Germany). After another centrifugation step (550*g*, 5 min, 4 °C), cells were again resuspended in a two-fold volume of swelling buffer and kept on ice for 20 min. Cells were mechanically lysed using a 2 ml Dounce homogenizer (Sartorius, Göttingen, Germany). Lysates were centrifuged (859*g*, 5 min, 4 °C) to remove cell nuclei and supernatants were mixed with one volume 2 × solubilization buffer (2% Triton X-100 in PBS) supplemented with protease inhibitor (Sigma-Aldrich). For membrane solubilization, lysates were rotated 30 min at 4 °C and centrifuged (1,700*g*, 15 min, 4 °C) to remove undissolved fragments. Myc-tagged Kv7.1 was purified using anti-myc 9E10 (kindly provided by Dr Ralf Trippe) coupled protein-A-sepharose beads (Sigma-Aldrich) or anti-myc agarose (Thermo Scientific, Darmstadt, Germany). For purification of KCNE1 anti-KCNE1 (N16) (sc-16796; Santa Cruz Biotechnology, Heidelberg, Germany) coupled protein-A-sepharose was used. For coupling of antibodies to protein-A-sepharose beads, 1 ml of each lysate was rotated with the respective antibody for 3 h before 30 μl (50% protein-A-sepharose) was applied. Anti-myc Agarose was added directly to the lysates. After overnight rotation at 4 °C, beads were seeded by centrifugation (425*g*, 2 min) and washed three times with 600 μl 1 × solubilization buffer. For MS-analysis, beads were resuspended in 25 mM AmBic (ammonium bicarbonate) and proteins were directly digested from the beads using trypsin protease. For Western blot analysis, purified proteins were eluted using 2 × Laemmli buffer. SDS–PAGE was performed using Mini-PROTEAN 3 or Mini-PROTEAN Tetra System (Bio-Rad Laboratories, Munich, Germany). Proteins were transferred to nitrocellulose membranes using Mini Trans-Blot or Trans-Blot Turbo (Bio-Rad Laboratories). Unspecific binding sites were blocked by incubation of membranes with 5% milk powder in TBS-T (TBS-T in mM: 20 Tris-HCl, 140 mM NaCl, 0.05% Tween). To detect Kv7.1myc, anti-myc 9E10 was used (3:10,000 in TBS-T). KCNE1 was detected using an antibody against KCNE1 (N16) (sc-16796; Santa Cruz Biotechnology, Heidelberg, Germany). Primary antibodies were incubated overnight at 4 °C. Before incubation with secondary antibodies, membranes were washed three times with TBS-T (5 min). The secondary antibodies used were horseradish peroxidase-conjugated anti-mouse (Amersham) and horseradish peroxidase-conjugated anti-goat (Santa Cruz Biotechnology). Before detection, membranes were washed four times with TBS-T (5 min) and two times with TBS (5 min). Super Signal West Pico kit was used for detection (Thermo Scientific).

### Mass spectrometry

Affinity purified Kv7.1 and KCNE1 proteins were digested from the beads with sequencing grade trypsin (Promega, Mannheim, Germany) overnight in the presence of 1% Rapigest. To quench digestion and degradation of Rapigest formic acid (acid-labile SDS-like detergent, (Waters Corporation, USA) was added. Samples were vacuum concentrated, reconstituted in 10 μl 2% acetonitrile (ACN) and 0.1% FA and loaded onto a self-packed 15 cm, 100 μm inner diameter Luna C18 reversed phase column (3 μm particles, 300 Å; Phenomenex, Aschaffenburg, Germany). Peptides were eluted into an LTQ Orbitrap Velos mass spectrometer (Thermo Fisher Scientific) via electrospray ionization (1.8 kV) using an Accela HPLC (Thermo Fisher Scientific) with an effective flow rate of approximately 250 nl min^−1^ and a 4 h ACN gradient from 5 to 85% ACN optimized for highly hydrophobic peptides. The 20 most intense precursors in the range from 400 to 2,000 *m/z* with charge stages from 2 to 4 were selected for MS/MS sequencing from a previous survey scan in the Orbitrap mass analyser, fragmented by collision induced dissociation and analysed in the linear ion trap mass analyser. Database searches were performed with the Proteome Discoverer software version 1.3 (Thermo Fisher Scientific) deploying the Sequest algorithm and searching against the human SwissProt database (release 15.6/57.6) allowing 10 ppm for precursor mass tolerance and 1.0 Da for fragment ion mass tolerance. Proteins were considered as present in the samples with at least 2 unique peptides with peptide false discovery rate <1% (based on decoy database) identified per peptide. The experiment was repeated three times with similar results. As a negative control, unlabelled channel proteins were analysed.

### Molecular modelling

A previously described 3D-model of the Kv7.1/KCNE1 channel was utilized for docking of AC-1 (JNJ303) and AC-10 (ref. 33). AC-1 was docked manually in proximity to the functionally identified key residues. The modelled Kv7.1/KCNE1-AC-1 complex was energy minimized using Yasara Structure 10. In a second approach, AC-10 was generated with a linker corresponding to the UV-reacted compound. The end of the linker (C25) was constrained to a distance of 2±1 Å to the oxygen OG1 in the KCNE1 Thr58 side chain. This positions the reacted linker in a distance to virtually allow for a similar covalent bond with both the side chain oxygen and the side chain carbogen of Thr58. A short all-atoms-mobile MD simulation using force field Amber03 (2 fs time steps) simulation was performed.

### Channel state modelling

The basic Markov model has been described before^35^. This Markov model was modified to recapitulate AC-1 effects best. The exact values are given in the figure legend.

### Data availability

The data that support the findings of this study are available from the corresponding author on reasonable request.

## Additional information

**How to cite this article:** Wrobel, E. *et al*. KCNE1 induces fenestration in the Kv7.1/KCNE1 channel complex that allows for highly specific pharmacological targeting. *Nat. Commun.* 7:12795 doi: 10.1038/ncomms12795 (2016).

## Supplementary Material

Supplementary Information Supplementary Figure 10 and Supplementary Table 1.

## Figures and Tables

**Table 1:** IC_50_ values for AC-1 determined for mutant Kv7.1/KCNE1 channels (*n*=3–11).

	IC_50_
Kv7.1 wt/KCNE1	78±1.2 nM
V241A/KCNE1	234±15 nM
I274A/KCNE1	327±83 nM
T312S/KCNE1	>50 μM
V334A/KCNE1	833±0.1 nM
F335A/KCNE1	171±0.02 nM
A336C/KCNE1	300±0.1 nM
I337V/KCNE1	6±1.6 μM
A344C/KCNE1	1±0.2 μM

IC_50_, half-maximum inhibitory concentration.
