# Supplementary material for: KCNE1 induces fenestration in the Kv7.1/KCNE1 channel complex that allows for highly specific pharmacological targeting
Source: Nat Commun. 2016 Oct 12;7:12795. doi: 10.1038/ncomms12795 (PMC5064022; doi:10.1038/ncomms12795)
Supplement: Supplementary Information — Supplementary Figure 10 and Supplementary Table 1. [file ncomms12795-s1.pdf]

Supplementary figures

Suppl. fig. 1

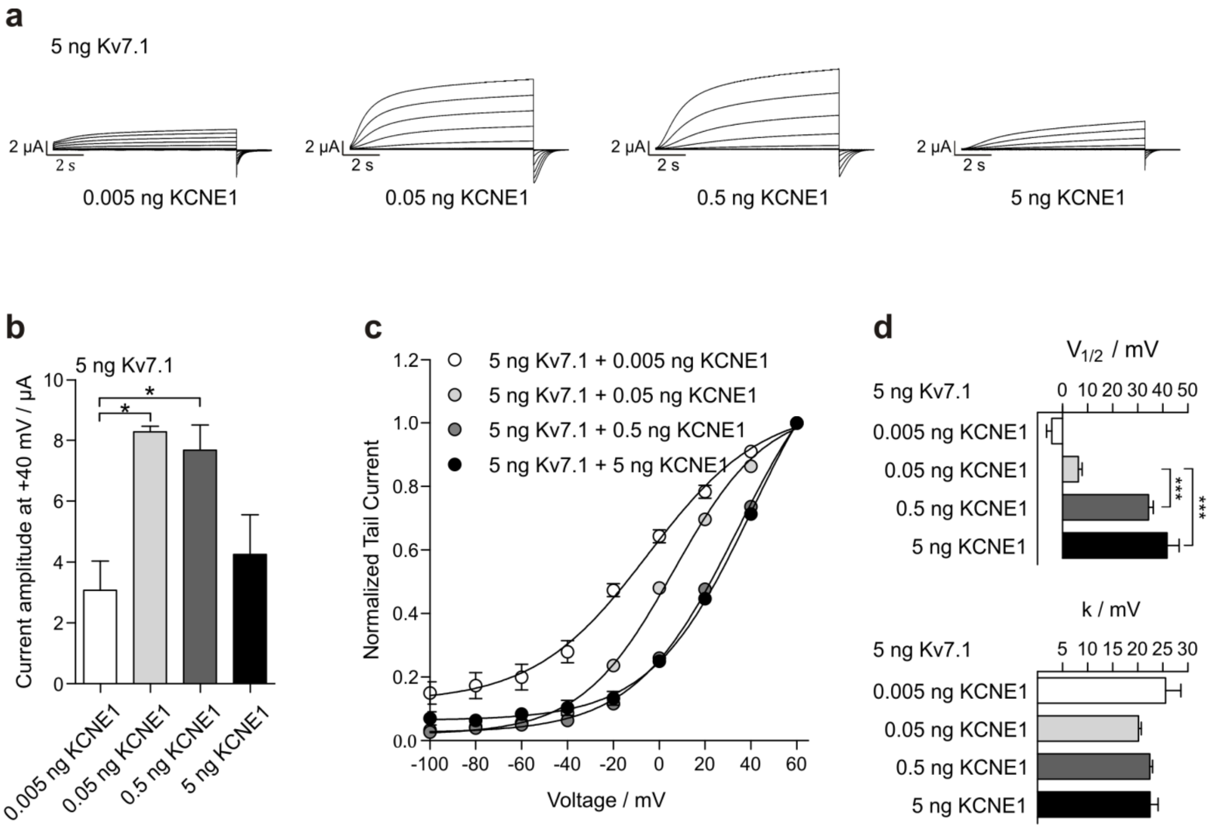

Supplementary Figure 1 | Influence of the level of KCNE1 expression on biophysical properties of Kv7.1 channels. (**a**) Example of currents conducted by channels formed in oocytes after injection of a fixed quantity of Kv7.1 cRNA (5 ng) and the indicated amounts of KCNE1. (**b**) Current amplitude at +40 mV varies with the amount of KCNE1 cRNA injected (*n* = 3, ± SEM; one way ANOVA with Dunnett's post hoc test; **P*<0.05). (**c**) Voltage dependence of activation is right shifted with increasing amounts of KCNE1 cRNA. Tail currents were recorded at -120 mV. Activation curves were obtained by plotting normalized initial tail currents against the voltage of the test pulse. Curves were fitted to a Boltzmann equation. (**d**) Voltage of half-maximal activation is significantly increased with increasing amounts of injected KCNE1 cRNA, but slope factor k was not significantly changed (*n*=3, ± SEM; one way ANOVA with Dunnett's post hoc test; ****P*<0.001).

Suppl fig. 2

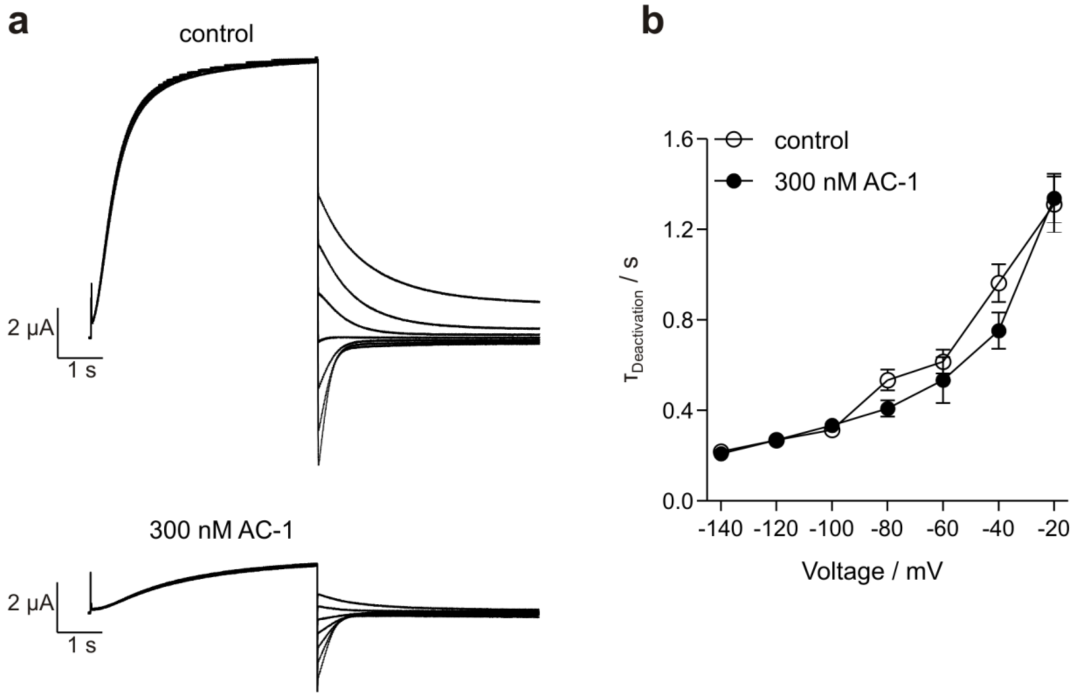

Supplementary Figure 2 | (**a**) Example of Kv7.1/KCNE1 current traces recorded before and after application of 300 nM AC-1. Currents were activated by a 5 s depolarizing pulse to +40 mV. Tail currents were recorded at different test potentials ranging from -140 mV to -20 mV, applied in 20 mV increments. (**b**) AC-1 does not alter the rate of current deactivation measured by fitting traces to a single exponential function to determine $\tau_{deactivation}$ (*n*=5, ± SEM; student's t-test).

Suppl fig. 3

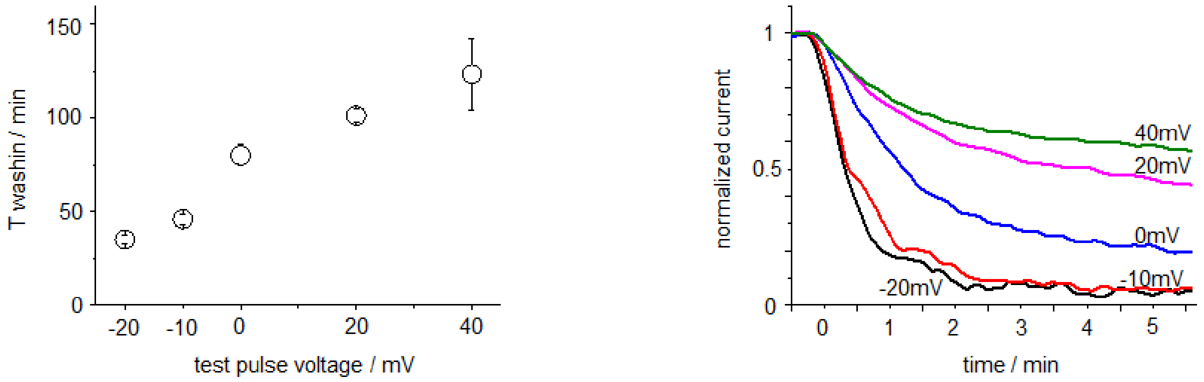

Supplementary Figure 3 | AC-1 binds to Kv7.1/KCNE1 channels in a closed state.

(Kv7.1/KCNE1 channels were activated by depolarizing pulses and 100 nM AC-1 was applied when channels were activated and reached steady state activation. Time constants for current inhibition (τ_{inhib}) were analyzed by fitting a single exponential function to the decrease in currents upon application of 100 nM AC-1 (n = 3-5). Representative current traces are shown normalized to the amplitude at steady state for test voltages of -20 mV (black); -10 mV (red); 0 mV (blue); 20 mV (magenta); 40 mV (green).

Suppl fig. 4

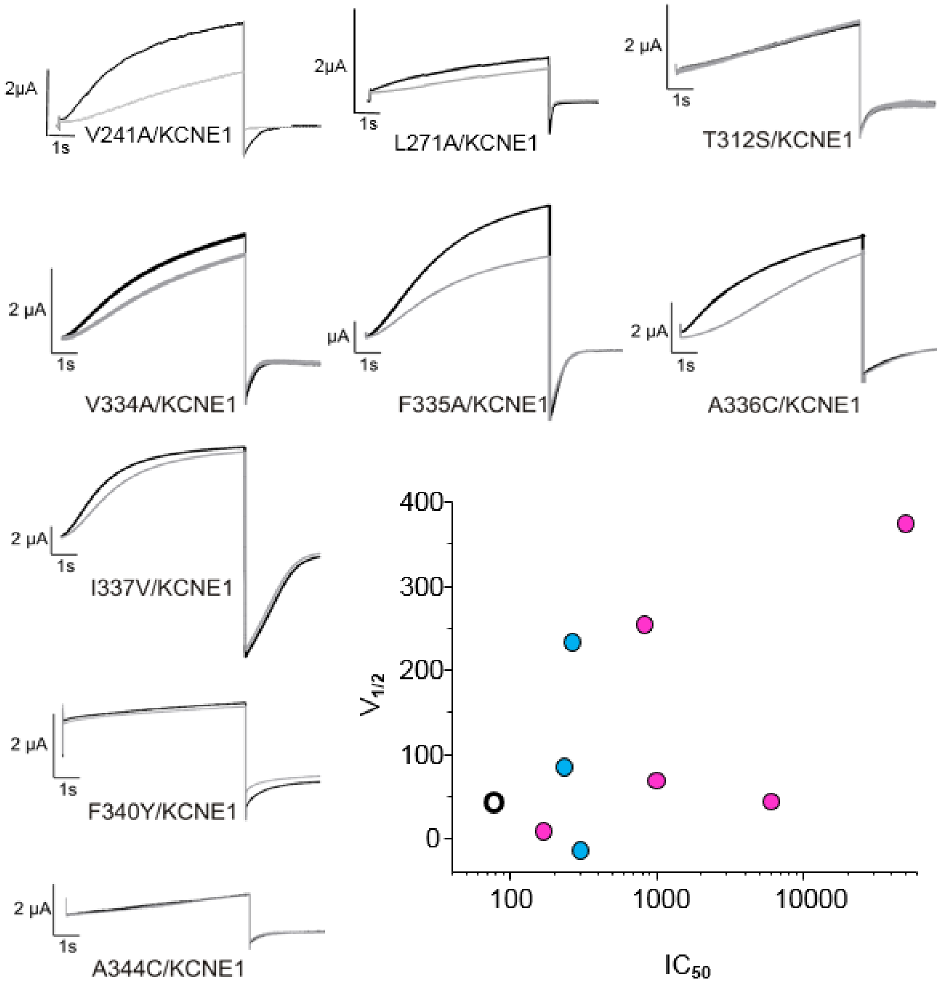

Supplementary Figure 4 | Kv7.1 mutations that alter the AC-1 sensitivity of Kv7.1/KCNE1 channels. Example of Kv7.1/KCNE1 current traces from channels harboring point mutations in Kv7.1 that were identified as crucial for AC-1 sensitivity. Graph lower right shows lack of correlation of IC_{50} vs. $V_{1/2}$. Data of fenestration facing residues in magenta filled, non-fenestration facing blue filled and wt in white filled circles.

Suppl fig. 5

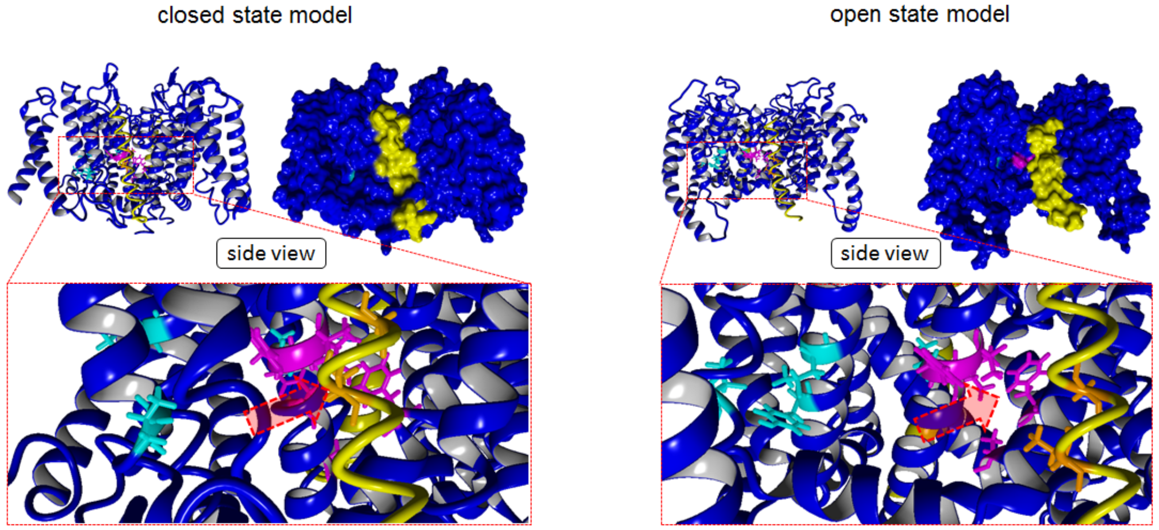

Supplementary Figure 5 | Lack of fenestration in closed and open state Kv7.1/KCNE1 channel models. Kv7.1/KCNE1 model complex in the closed and open state are shown. Ribbon representation with Kv7.1 in blue and KCNE1 in yellow. Key residues identified in the mutagenesis scan are colored as in Fig. 3 for better comparison. The whole Kv7.1/KCNE1 channel complex was surface rendered (upper right) but no fenestration is present in either of the two models. Approximate position of the fenestrations present in the preopen closed state model (**Fig. 3, 8**) is indicated by red arrows.

Suppl fig. 6

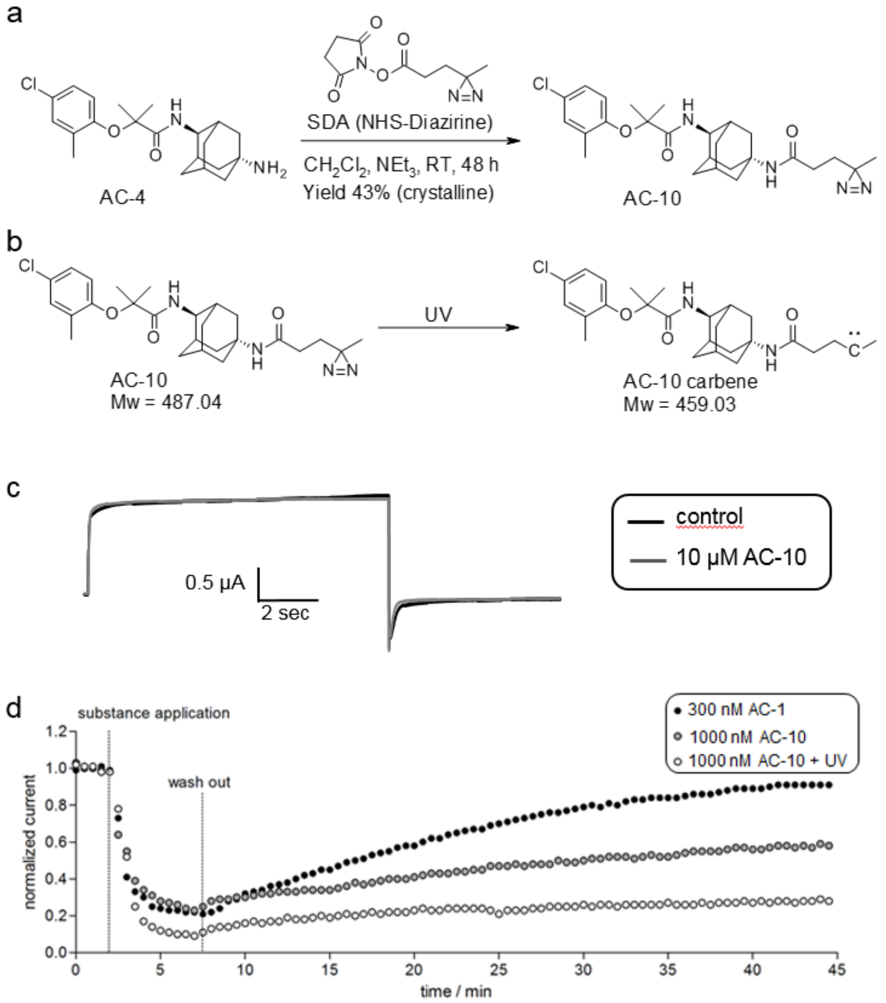

Supplementary Figure 6 |Synthesis, UV-induced modification of AC-10 and lack of effect on Kv7.1 without KCNE1. (**a**) AC-10 was synthesized by coupling an HNS-diazirine to the amino group of AC-4. (**b**) Diazirines can be activated by UV irradiation at 350 nm. UV activation of AC-10 generates a highly reactive carbene species that can covalently bind to all natural amino acids. (**c**) Kv7.1 expressed without KCNE1 was activated by a 20 mV pulse and 10 µM AC-10 was applied. Representative current traces are shown. (**d**) Washin and washout of AC-1, and AC-10 without UV irradiation or without.

Suppl fig. 7

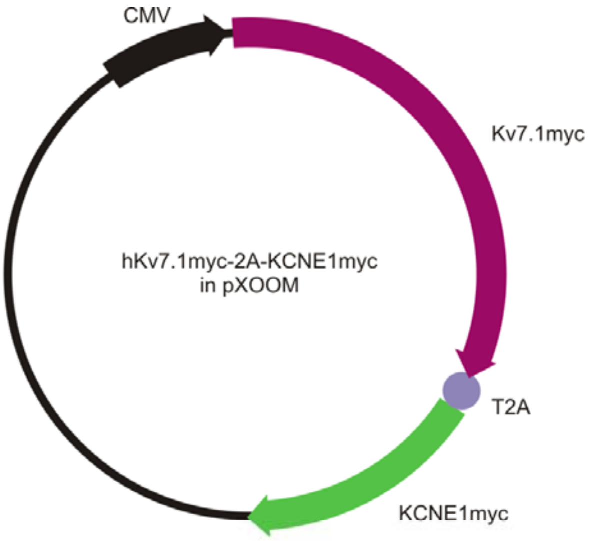

coding sequence: Kv7.1myc-2A-KCNE1myc

```
ATGGCCGCGGCCTCCTCCCCGCCCAGGGCCGAGAGGAAGCGCTGGGGTTGGGGCCGCCTGCCAGGCGCCCGGCGGGGCAGCGCGGG
CCTGGCCAAGAAGTGCCCCTTCTCGCTGGAGCTGGCGGAGGGCGGCCCGGCGGGCGGCGCGCTCTACGCGCCCATCGCGCCCGGCGC
CCCAGGTCCCGCGCCCCCTGCGTCCCCGGCCGCGCCCGCCGCGCCCCCAGTTGCCTCCGACCTTGGCCCGCGGCCGCCGGTGAGCCT
AGACCCGCGCGTCTCCATCTACAGCACGCGCCGCCCGGTGTTGGCGCGCACCCACGTCCAGGGCCGCGTCTACAACTTCCTCGAGCGTC
CCACCGGCTGGAAATGCTTCGTTTACCACTTCGCCGTCTTCCTCATCGTCCTGGTCTGCCTCATCTTCAGCGTGCTGTCCACCATCGAGCA
GTATGCCGCCCTGGCCACGGGGACTCTCTTCTGGATGGAGATCGTGCTGGTGGTGTTCTTCGGGACGGAGTACGTGGTCCGCCTCTGGT
CCGCCGGCTGCCGCAGCAAGTACGTGGGCCTCTGGGGGCGGCTGCGCTTTGCCCGGAAGCCCATTTCCATCATCGACCTCATCGTGGTC
GTGGCCTCCATGGTGGTCCTCTGCGTGGGCTCCAAGGGGCAGGTGTTTGCCACGTCGGCCATCAGGGGCATCCGCTTCCTGCAGATCCT
GAGGATGCTACACGTCGACCGCCAGGGAGGCACCTGGAGGCTCCTGGGCTCCGTGGTCTTCATCCACCGCCAGGAGCTGATAACCACCC
TGTACATCGGCTTCCTGGGCCTCATCTTCTCCTCGTACTTTGTGTACCTGGCTGAGAAGGACGCGGTGAACGAGTCAGGCCGCGTGGAGT
TCGGCAGCTACGCAGATGCGCTGTGGTGGGGGGTGGTCACAGTCACCACCATCGGCTATGGGGACAAGGTGCCCCAGACGTGGGTCGG
GAAGACCATCGCCTCCTGCTTCTCTGTCTTTGCCATCTCCTTCTTTGCGCTCCCAGCGGGGATTCTTGGCTCGGGGTTTGCCCTGAAGGTG
CAGCAGAAGCAGAGGCAGAAGCACTTCAACCGGCAGATCCCGGCGGCAGCCTCACTCATTCAGACCGCATGGAGGTGCTATGCTGCCGA
GAACCCCGACTCCTCCACCTGGAAGATCTACATCCGGAAGGCCCCCCGGAGCCACACTCTGCTGTCACCCAGCCCCAAACCCAAGAAGT
CTGTGGTGGTAAAGAAAAAAAAGTTCAAGCTGGACAAAGACAATGGGGTGACTCCTGGAGAGAAGATGCTCACAGTCCCCCATATCACGTG
CGACCCCCCAGAAGAGCGGCGGCTGGACCACTTCTCTGTCGACGGCTATGACAGTTCTGTAAGGAAGAGCCCAACACTGCTGGAAGTGA
GCATGCCCCATTTCATGAGAACCAACAGCTTCGCCGAGGACCTGGACCTGGAAGGGGAGACTCTGCTGACACCCATCACCCACATCTCAC
AGCTGCGGGAACACCATCGGGCCACCATTAAGGTCATTCGACGCATGCAGTACTTTGTGGCCAAGAAGAAATTCCAGCAAGCGCGGAAGC
CTTACGATGTGCGGGACGTCATTGAGCAGTACTCGCAGGGCCACCTCAACCTCATGGTGCGCATCAAGGAGCTGCAGAGGAGGCTGGAC
CAGTCCATTGGGAAGCCCTCACTGTTCATCTCCGTCTCAGAAAAGAGCAAGGATCGCGGCAGCAACACGATCGGCGCCCGCCTGAACCGA
GTAGAAGACAAGGTGACGCAGCTGGACCAGAGGCTGGCACTCATCACCGACATGCTTCACCAGCTGCTCTCCTTGCACGGTGGCAGCAC
CCCCGGCAGCGGCGGCCCCCCCAGAGAGGGCGGGGCCCACATCACCCAGCCCTGCGGCAGTGGCGGCTCCGTCGACCCTGAGCTCTT
CCTGCCCAGCAACACCCTGCCCACCTACGAGCAGCTGACCGTGCCCAGGAGGGGCCCCGATGAGGGGTCCGAACAAAAACTCATCTCAG
AAGAGGATCTGGGCTCCGGCGAGGGCAGGGGAAGTCTTCTAACATGCGGGGACGTGGAGGAAAATCCCGGCCCAATGATCCTGTCTAAC
ACCACAGCGGTGACGCCCTTTCTGACCAAGCTGTGGCAGGAGACAGTTCAGCAGGGTGGCAACATGTCGGGCCTGGCCCGCAGGTCCC
CCCGCAGCAGTGACGGCAAGCTGGAGGCCCTCTACGTCCTCATGGTACTGGGATTCTTCGGCTTCTTCACCCTGGGCATCATGCTGAGCT
ACATCCGCTCCAAGAAGCTGGAGCACTCGAACGACCCATTCAACGTCTACATCGAGTCCGATGCCTGGCAAGAGAAGGACAAGGCCTATG
TCCAGGCCCGGGTCCTGGAGAGCTACAGGTCGTGCTATGTCGTTGAAAACCATCTGGCCATAGAACAACCCAACACACACCTTCCTGAGA
CGAAGCCTTCCCCAGAACAAAAACTCATCTCAGAAGAGGATCTGStop
```

Supplementary Figure 7 | cDNA-construct used for expression of Kv7.1myc-2A-KCNE1myc channels in mammalian cells. (**a**) Vector map of Kv7.1myc-2A-KCNE1myc. (**b**) Sequence of the Kv7.1myc-2A-KCNE1 cDNA construct for mammalian expression.

Suppl fig. 8

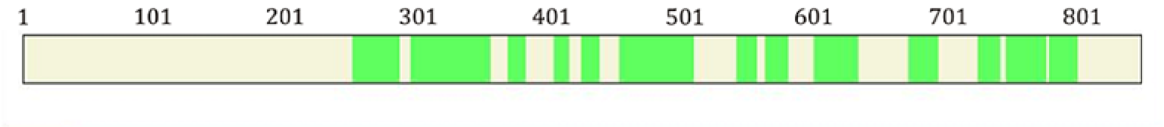

Kv7.1*myc* (1-686)

1 MAAASSPPRAERKRWGWGRLPGARRGSAGLAKKCPFSLELAEGGPAGGALYAPIAPGAPGPAPPASPAAPAAPPVASDLGPRPPVSLDPRVSIYSTRRPV

S1-Segment S2-Segment

101 LARTHVQGRVYNFLERPTGWKCFVYHFAVFLIVLVCLIFSVLSTIEQYAALATGTLFWMEIVLVVFFGTEYVVRLWSAGCRSKYVGLWGRLRFARKPISI

S3-Segment S4-Segment S5-Segment

201 IDLIVVVASMVVLCVGSKGQVFATSAIRGIRFLQILRMLHVDRQGGTWRLLGSVVFIHRQELITTLYIGFLGLIFSSYFVYLAEKDAVNESGRVEFGSYA

Pore S6-Segment

301 DALWWGVVTVTTIGYGDKVPQTWVGKTIASCFSVFAISFFALPAGILGSGFALKVQQKQRQKHFNRQIPAAASLIQTAWRCYAAENPDSSTWKIYIRKAP

401 RSHTLLSPSPKPKKSVVVKKKKFKLDKDNGVTPGEKMLTVPHITCDPPEERRLDHFSVDGYDSSVRKSPTLLEVSMPHFMRTNSFAEDLDLEGETLLTPI

501 THISQLREHHRATIKVIRRMQYFVAKKKFQQARKPYDVRDVIEQYSQGHLNLMVRIKELQRRLDQSIGKPSLFISVSEKSKDRGSNTIGARLNRVEDKVT

c-myc T2A (689-707)

601 QLDQRLALITDMLHQLLSLHGGSTPGSGGPPREGGAHITQPCGSGGSVDPELFLPSNTLPTYEQLTVPRRGPDEGSEQKLISEEDLGSGEGRGSLLTCGD

KCNE1*myc* (708-846) transmembrane helix

701 VEENPGPMILSNTTAVTPFLTKLWQETVQQGGNMSGLARRSPRSSDGKLEALYVLMVLGFFGFFTLGIMLSYIRSKKLEHSNDPFNVYIESDAWQEKDKA

c-myc

801 YVQARVLESYRSCYVVENHLAIEQPNTHLPETKPSPEQKLISEEDL

Supplementary Figure 8 | Coverage of MS/MS. Peptides identified by MS/MS form mammalian cells expressed with the construct described in supplementary Fig. 7 are colored green.

Suppl fig. 9

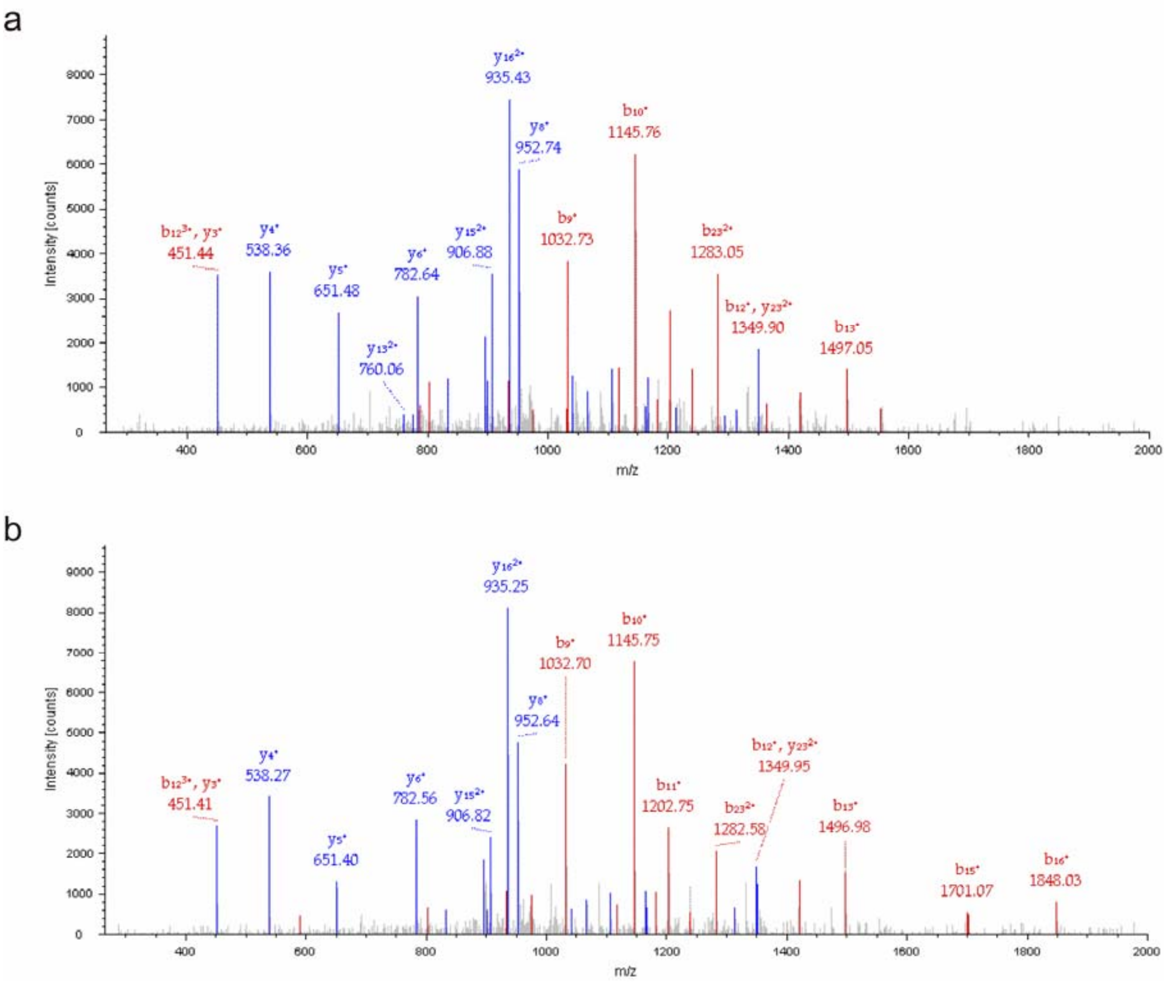

Supplementary Figure 9 | KCNE1 modification by AC-10 is not detected in the absence of Kv7.1 and not in coexpression with Kv2.1. MS/MS spectra of the triply charged species of the non-modified KCNE1 peptide ^{42}LEALYVLMVLGFFGFFTLGIMLSYIR^{67} originating from KCNE1 **(a)** and Kv2.1-KCNE1 **(b)**. Consecutive ion series are observed with b- (red) and y-ion annotation (blue). Beside excellent database search criteria (XCorr > 5.6) our results are strengthened by the point that relative ion intensities are similar in both spectra.

Suppl fig. 10

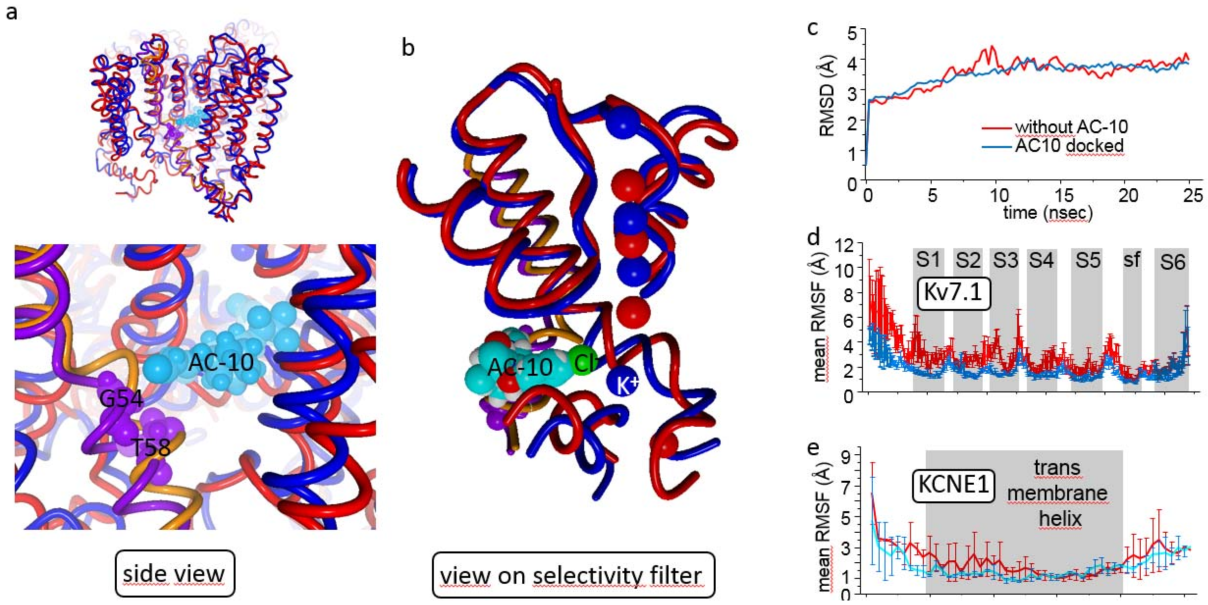

Supplementary Figure 10 | Molecular dynamics simulation of Kv7.1/KCNE1 in presence and absence of AC-10 (**a**) AC-10 was docked in close proximity to allow for (covalent) binding to g54 and T58. A 25 nsec all atoms mobile simulation was performed with one AC-10 in the channel complex using standard approach "run-membrane.mcr" build in YasarStructure14.7.17. A second parallel simulation with identical parameter settings but without AC-10 was conducted. AC-10 stayed very stable in its proposed binding site during the complete simulation. The average positions of residues were calculated and are shown in (a,b). One side in proximity to KCNE1 residues G54 and T58 (**a**) and on the other side a coordinated a K^+ ion in the central cavity (**b**) during the simulation. (**c**) after about 10 nsec both simulations were in equilibrium as suggested by the RMSD calculations. (**d,e**) the model containing AC-10 was much more stable during the simulation compared to the simulations without AC-10. Fluctuations per residue were calculated and mean +/- SEM were calculated for Kv7.1 or for KCNE1 subunits. Positions of the transmembrane helices and the selectivity filter (sf) are indicated by gray bars.

Supplementary table

Suppl. table 1 | SAR and physical properties of AC derivatives

SAR and physical properties of AC derivatives (n=3-13).

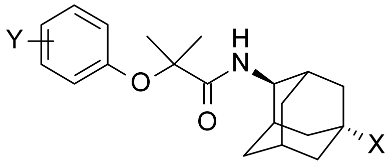

Substance	Substituent X	Substituent Y	M_w /Da	Volume /cm^3	miLogP	Inhibition by 10 µM / %
AC-1	-$NHSO_2CH_3$	4-Cl	440.98	330.5	4.0	89.2 ± 1.2
AC-2	-$NHSO_2CH_3$	2-CH_3,4-Cl	455.01	346.2	4.4	86.8 ± 3.0
AC-3	-$NHSO_2CH_3$	3-CF_3	474.54	350.5	4.2	73.7 ± 2.9
AC-4	-NH_2	2-CH_3,4-Cl	376.92	307.0	4.7	4.2 ± 6.1
AC-5	-OH	2-CH_3,4-Cl	377.90	300.8	4.6	40.9 ± 1.4
AC-6	-COOH	2-CH_3,4-Cl	405.92	313.8	4.5	64.6 ± 6.6
AC-7	-$NHSO_2N(CH_3)_2$	2-CH_3,4-Cl	484.05	368.6	4.4	38.0 ± 4.4
AC-8	-$NHCOOCH_2CH_3$	2-CH_3,4-Cl	448.98	362.3	5.5	74.5 ± 6.2
AC-9	-$NHCOCH_2CH(CH_3)_2$	2-CH_3,4-Cl	461.04	389.1	6.0	78.6 ± 3.3
